# Multiple Pathways of Visual Adaptations for Water Column Usage in an Antarctic Adaptive Radiation

**DOI:** 10.1002/ece3.70867

**Published:** 2025-03-09

**Authors:** Ella B. Yoder, Elyse Parker, Bruno Frédérich, Alexandra Tew, Christopher D. Jones, Alex Dornburg

**Affiliations:** ^1^ Department of Bioinformatics and Genomics University of North Carolina at Charlotte Charlotte North Carolina USA; ^2^ Research Triangle High School Durham North Carolina USA; ^3^ Department of Ecology and Evolutionary Biology Yale University New Haven Connecticut USA; ^4^ Laboratory of Evolutionary Ecology, FOCUS University of Liège Liège Belgium; ^5^ Ecosystem Science Division NOAA Southwest Fisheries Science Center La Jolla California USA

**Keywords:** icefish, notothenioids, opsin, tuning site

## Abstract

Evolutionary transitions in water column usage have played a major role in shaping ray‐finned fish diversity. However, the extent to which vision‐associated trait complexity and water column usage is coupled remains unclear. Here we investigated the relationship between depth niche, eye size, and the molecular basis of light detection across the Antarctic notothenioid adaptive radiation. Integrating a phylogenetic comparative framework with data on eye size and depth occupancy, we provide support for an acceleration in the rate of eye size diversification nearly 20 million years after the initial radiation. Our results further reveal that levels of eye size divergence are often highest between closely related taxa. We further analyzed opsin tuning site sequences and found changes representing repeated instances of independent tuning site changes across the notothenioid phylogeny that are generally not associated with habitat depth or species eye size. Collectively, our results strongly support that multiple evolutionary pathways underlie the diversification of visual adaptations in this iconic adaptive radiation.

## Graphical abstract

By investigating the diversification of the visual system in notothenioids, we reveal lineages at equivalent depths often depict extremes in eye size with striking convergences in tuning sites between distantly related species that are likely driven by the unusual environmental conditions of the Southern Ocean. This finding represents a striking departure from the expectations of visual diversification in other aquatic lineages and raises new research questions that simultaneously challenge prevailing models of neural diversification while offering a new perspective on visual diversification during adaptive radiation.

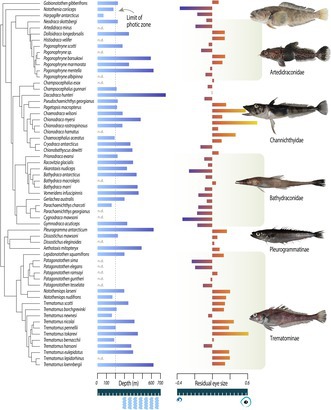

## 1 Introduction

Evolutionary changes in water column usage have shaped the diversification of aquatic organisms across the Tree of Life (Modica et al. 2020; Rosa et al. 2008). The evolutionary history of ray‐finned fishes (Actinopterygii) provides numerous striking examples of transitions between benthic and pelagic habitat or depth usage that are associated with the diversification of morphological (Ingram 2011; John et al. 2022; Smith and Brown 2002), physiological (Brownstein et al. 2024; Brown and Thatje 2014), or ecological traits (Costello and Chaudhary 2017; Fukunaga et al. 2016). Such diversification is readily apparent when considering the sometimes extreme heterogeneity in the variation of traits such as eye size that are associated with vision (Caves, Sutton, and Johnsen 2017; Goatley and Bellwood 2009). Shifts in available light across the water column pose a challenge to fundamental aspects of the ecology of ray‐finned fishes including finding food or mates, navigating around complex structures, and avoiding predators (de Busserolles et al. 2020; Warrant 2000). Shifts in eye size provide a means for surmounting these challenges by changing the focal length (Kröger, Fritsches, and Warrant 2009; Warrant and Locket 2004), pupil area (MacIver et al. 2017; Wagner et al. 1998), and distribution of photoreceptors (de Busserolles and Marshall 2017). In turn, these modifications promote changes in acuity (Caves, Sutton, and Johnsen 2017) and the sensitivity of the eye (de Busserolles and Marshall 2017; Warrant 2000, 2004; Warrant, Collin, and Locket 2008). For example, morphological and processing adaptations associated with larger eyes can aid in detecting approaching predators or prey in dim light (Kotrschal et al. 2017; Nilsson et al. 2012; Vinterstare et al. 2020). Consequently, dramatic shifts in eye size are common between diurnal and nocturnal species, as well as between shallow, diel‐migrating, and deep‐dwelling taxa, as downwelling light diminishes with depth (de Busserolles et al. 2014; de Busserolles and Marshall 2017; Hunt et al. 2015; Schmitz and Wainwright 2011; Sutton 2013). However, in some taxa, evolutionary pressures have favored alternative adaptations that minimize visual investment (e.g., chemoreception, hearing, lateral line systems (Blin et al. 2024; Marranzino and Webb 2018; Ruppé et al. 2015)).

Minimizing photoreception may represent a response to a tradeoff in neural investment between photo and chemoperception (Iglesias et al. 2018) that is especially relevant for polar fishes. These fishes experience prolonged darkness for half of the year and intense seasonal light during the other half. Species in these environments may therefore be expected to have reduced eye sizes that would limit the perceivable visual spectrum, and reallocate neural resources to enhance chemical detection, which remains useful in dark conditions (Kuball et al. 2024). However, many fishes found in the water column at polar latitudes possess large eyes. This suggests that these lineages are under selection to optimize specific photoreceptors to function across a limited, consistent spectral range year‐round. For example, retaining a range of retinal cone types (i.e., photoreceptor cells) tuned to blue and UV wavelengths (Pointer et al. 2005) can enable discriminating objects against a light background color, aiding in both predator avoidance or prey capture (Lythgoe et al. 1994; Marshall, Carleton, and Cronin 2015). Such a scenario suggests that diversification of the visual system may be more constrained by the light environment than the diversification of other aspects of ecomorphology (Ricci et al. 2023). However, this hypothesis remains untested in polar waters.

Cryonotothenioids, the adaptive radiation of notothenioids in the Antarctic, represent an exemplar system for investigating the diversification of the visual system across water‐column niches. This clade of approximately 100 species (Eastman and Eakin 2021) dominates near‐shore Antarctic waters, serving as essential food sources for many predators, including penguins, seals, and whales (La Mesa, Dalú, and Vacchi 2004), and supporting a significant commercial fishery (Abrams 2014; Constable et al. 2000). A rare example of adaptive radiation in a marine environment (Daane et al. 2019; Eastman 2000; Matschiner, Hanel, and Salzburger 2011), cryonotothenioids have diversified into water column niches spanning hundreds of meters in depth, largely driven by ecological opportunities following cyclical ice‐scouring events that periodically decimate benthic communities across the Antarctic continental shelf (Dornburg et al. 2017; Near et al. 2012; Strugnell et al. 2012; Thatje, Hillenbrand, and Larter 2005; Thatje et al. 2008). This pattern of cyclical annihilation and recovery has promoted divergence among closely related lineages, resulting in marked variability in their water‐column niches (Parker et al. 2022). Alongside their diversification across the water column, cryonotothenioids exhibit dramatic variation in eye size, encompassing a level of diversity nearly as high as that exhibited collectively across all the near‐shore coastal fishes of New Zealand (Montgomery and Macdonald 1998). However, the mechanisms promoting the diversification of eye sizes in cryonotothenioids remains unexplored, as does the evolutionary relationship between eye size and light detection.

At the molecular level, the process of vision is initiated through a light‐induced conformational change in opsins, photopigments which are bound to a vitamin A‐derived chromophore (Terakita 2005). Phototransduction occurs through the interaction between opsin protein and the chromophore, which determines the maximum spectrum absorbance (*λ*_max_) of the opsin. A number of residues have been identified in opsins as “tuning sites” that influence which wavelengths (*λ*_max_) trigger phototransduction and the wavelengths of detectable light (Musilova, Salzburger, and Cortesi 2021). Across vertebrates, rods contain only one class of opsin (rhodopsin‐like‐1; Rh1) that is responsible for scotopic (dim‐light) vision (Pisani et al. 2006; Yokoyama et al. 2008). Cones contain four classes of opsins that are responsible for photopic (bright‐light) vision. Short‐wavelength‐sensitive‐1 (SWS1) and short‐wavelength‐sensitive‐2 (SWS2) absorb in the ultraviolet (UV; peak spectral sensitivity [*λ*_max_] = 355–450 nm) and violet/blue (*λ*_max_ = 415–490 nm) regions of the spectrum, respectively. Middle‐wavelength‐sensitive/rhodopsin‐like‐2 (Rh2) is most sensitive to the central (green) waveband (*λ*_max_ = 470–535 nm), and long wavelength sensitive (LWS) is tuned toward the spectrum's red end (*λ*_max_ = 490–570 nm) (Yokoyama 2002). Early investigations of cryonotothenioid opsins suggested that these lineages possess a limited repertoire of opsins relative to other ray‐finned fishes (Pointer et al. 2005). While a reduction of opsins could be expected for organisms living with seasonal light availability at varying depths, it is now clear that this is not the case. Instead, cryonotothenioid opsin diversity is on par with that of other fishes possessing complex color tetrachromatic vision (Hunt et al. 2001; Lin et al. 2017; Musilova, Salzburger, and Cortesi 2021; Rennison, Owens, and Taylor 2012).

The finding of possible tetrachromatic vision raises the question of how opsin tuning sites evolved during the radiation of cryonotothenioids. On one hand, eye size may be modulated by changes in depth usage and associated with specific tuning site changes toward the detection of shorter wavelengths. Such a hypothesis would align with expectations of selective pressure exerted by depth‐specific photic conditions. On the other hand, eye size may be conserved over short evolutionary timescales (de Busserolles et al. 2013) and could be decoupled from changes in tuning sites occurring as lineages transition to alternate water‐column niches. This second hypothesis suggests that eye size variation among cryonotothenioids may reflect phylogenetic inertia rather than direct selection on visual traits for specific depths. The degree to which these or other hypotheses explain the evolutionary dynamics of eye size and opsin tuning in cryonotothenioids remains unresolved.

Here we investigate the evolutionary history of eye size and opsin tuning sites across cryonotothenioids using a time calibrated phylogenetic framework. We analyzed eye size data collected from museum specimens, two decades of National Oceanographic and Atmospheric Administration (NOAA) trawl‐based surveys, and data on buoyancy in a series of comparative phylogenetic analyses to test the associations between these traits and depth. We then tested whether cryonotothenioids show signatures of an early burst of diversification in eye size and depth, which would indicate strong initial selection across depth zones during the onset of adaptive radiation. Finally, we assess molecular adaptation by analyzing opsins from all major cryonotothenioid lineages using publicly available genome and transcriptome data, as well as sequences from NCBI (National Center for Biotechnology Information). Collectively, the results of our analyses reveal unusual patterns of water‐column and vision‐associated diversification of these traits, providing new insights into how cryonotothenioids' visual systems evolved in response to Antarctic water‐column niche specialization.

## 2 Methods

### 2.1 Collection of Morphological and Ecological Data

We assessed patterns of diversification in cryonotothenioid eye size and investigated the extent to which evolutionary changes in eye size are modulated by diversification along the water column. We used the program TpsDIG2 (Rohlf 2006) to quantify eye size and body size from digital images of 161 specimens from museum specimens (Table S1) representing 61 cryonotothenioid species spanning all major notothenioid lineages (Near et al. 2018; Table S1). Where possible, we sampled three adult individuals per species that exhibited a range of variation in body size, but in some cases only one or two specimens were available. Eye diameter was measured from the most anterior point of the eye to the most posterior point of the eye. Body size was represented using standard length (SL), measured as the distance from the tip of the snout to the posterior end of the hypural plate. We additionally measured maximum body depth as the maximum distance between the outer dorsal and outer ventral surfaces and head length as the distance from the tip of the snout to the posterior edge of the operculum.

We then compiled data on water column usage for the same species measured in our morphological dataset. Depth occupancy was represented using three different measures: (1) mean depth of capture calculated for 44 of our 61 focal species calculated from over 19,900 trawl records of adult specimens from the U.S. Antarctic Marine Resources Program (U.S. AMLR) Antarctic finfish surveys between 1998 and 2018; (2) minimum reported depth occupancy for all 61 focal species (summarized in Eastman 2017); and (3) maximum reported depth occupancy for all 61 focal species (summarized in Eastman (2017)). We incorporated the minimum and maximum reported depths to account for the potential bias introduced in our analysis by the limited depth range recorded during AMLR bottom‐trawl survey protocols. Specifically, the records used to quantify mean depth were obtained from trawls conducted primarily within the 500 m isobath of the South Orkney Islands and Antarctic Peninsula shelf and slope (Balguerias 2000; Jones et al. 2009). However, many notothenioid species are known to occupy bathymetric distributions extending to depths of 1000 m or more (Eastman 2017), and average depth occupancy is likely to vary across different regions of the Antarctic continental shelf. Our inclusion of minimum‐ and maximum‐recorded depth variables therefore allowed us to more fully capture the range of variation in depth occupancy across our focal notothenioids.

For 46 of our focal species, we additionally represented water column niche using data on mean percentage buoyancy (%B), which is a measure of the overall body density of species calculated as the percentage of body weight in air supported in seawater (Eastman 2020; Eastman and DeVries 1982; Near et al. 2012). All cryonotothenioids lack a swim bladder, and their repeated diversification into habitats along the benthic‐pelagic axis of the water column has been achieved by the evolution of reduced skeletal ossification and lipid deposits that alter these fishes' relative weights in seawater (Daane et al. 2019; Daane and William Detrich III 2021). As such, %B can be used as a proxy for water column niche use in cryonotothenioids (Eastman 2020). To avoid the potential of ontogenetic allometry skewing results, only data from adult fishes was used for all analyses.

### 2.2 Calculating the Impact of Water Column Usage and Buoyancy on Eye Size

We used time‐calibrated phylogenies taken from Parker et al. (2022) to conduct a series of comparative phylogenetic analyses that evaluated the relationships among evolutionary changes in water column usage and diversification of eye size in cryonotothenioids. Measurements of morphological traits are expected to scale with measures of body size (Berner 2011), and eye diameter is correlated with body length, body depth, or head length in cryonotothenioids. As such, we represented eye size using residuals from a phylogenetic generalized least squares (PGLS) regression (implemented using the gls function in the nlme v3.1 R package Pinheiro and Bates 2000) of eye diameter on standard length. This approach enables us to fit linear models of trait correlation while also accounting for the covariance among traits due to phylogeny. For all tests, we used the corBrownian function in the ape v5.7 R package (Paradis, Claude, and Strimmer 2004) to define the trait covariance structure using a Brownian motion (BM) model of trait evolution on the time calibrated phylogeny of cryonotothenioids. To assess the sensitivity of our results to the choice of evolutionary model, all analyses were repeated using an Ornstein‐Uhlenbeck (OU) model of trait evolution. As head length (HL) represents an alternate measure of body size sometimes used in studies of fishes, we additionally repeated all downstream comparative phylogenetic analyses representing eye size using residuals from the regression of eye diameter on HL. This allowed us to assess the sensitivity of our results to measures of body size. Finally, to determine whether depth niche (benthic and demersal) is associated with differences in eye size while accounting for phylogenetic relationships, we conducted a phylogenetic ANCOVA with depth niche expressed as a categorical factor, following the approach in Foster et al. (2018).

We visually assessed the relationships among eye size, depth occupancy, and buoyancy across the time‐calibrated cryonotothenioid phylogeny using a series of phenograms generated in the R package phytools v0.2.2 (Revell 2012). These projections of a phylogeny in trait space have branch lengths corresponding to evolutionary time and the vertical positions of the tips corresponding to measured trait values. We used the contMap function of phytools to map evolution of mean depth of occurrence onto the projected phylogeny in order to simultaneously visualize patterns of evolution in eye size and depth occupancy along the cryonotothenioid phylogeny. This procedure was repeated with %B mapped onto a phylogeny projected into trait space defined by eye size in order to compare evolutionary patterns of eye size and buoyancy.

We then calculated relative subclade disparity through time (DTT; Harmon et al. 2003) to evaluate patterns of ecological and morphological diversity over the course of the cryonotothenioid radiation using the R package geiger2 v2.0.11 (Pennell et al. 2014) for the traits of eye size, mean depth of occurrence, and %B. Briefly, this approach provides a quantification of the disparity within and between clades over the temporal history of a phylogeny. High disparity values indicate extensive within‐subclade variation, indicating high levels of divergence among close relatives. In contrast, low values indicate that most variation is partitioned among distinct subclades (Harmon et al. 2003). We assessed whether patterns of cryonotothenioid trait disparity significantly departed from expectations under a null model of Brownian motion generated from 10,000 simulations of trait evolution on the cryonotothenioid phylogeny. Divergence of the empirical DTT curve from the Brownian motion simulations was assessed using the morphological disparity index (MDI), with negative MDI values indicating that trait disparity is partitioned mostly among subclades, while positive MDI values suggest that trait disparity is partitioned within subclades. Following Harmon et al. (2003), we restricted our calculation of MDI values to only the first 80% of the cryonotothenioid time tree to reflect our taxon sampling not capturing all tipward divergences. We complemented our DTT analysis by evaluating the fit of five different models of eye size evolution across the notothenioid phylogeny. We applied the fitContinuous function from the R package geiger2 to compare the fit of Brownian motion (BM), Ornstein–Uhlenbeck (OU), early burt (EB), white noise, and delta models using a sample size‐corrected modification of the Akaike Information criterion (AICc). To differentiate model fit, we used a ∆AICc threshold of two in combination with a calculation of Akaike weight of each model to determine the best‐fitting model.

In addition to those two qualitative descriptions of the evolution of eye size, depth occupancy, and buoyancy, we aimed to evaluate the extent to which variation in our focal ecomorphological traits can be explained by phylogeny. Thus, we calculated two measures of phylogenetic signal in eye size, mean depth of occurrence, and mean %B. Specifically, we used the phylosig function implemented in the R package phytools (Revell 2012) to calculate Pagel's *λ* (Pagel 1999) and Blomberg et al.'s *K* (Blomberg, Garland Jr., and Ives 2003) for each trait based on the time‐calibrated cryonotothenioid phylogeny. For Pagel's *λ*, a value of zero indicates no phylogenetic signal is present in the data, and a value of one indicates that phylogenetic signal is consistent with expectations of a BM model of trait evolution. The statistical significance of Pagel's *λ* values was determined using a likelihood ratio test comparing the empirical value of Pagel's *λ* to the null expectation that *λ* = 0. For Blomberg's *K*, values less than one suggest lower phylogenetic signal than expected under a BM model, while values greater than one suggest higher than expected phylogenetic signal. The statistical significance of Blomberg's *K* was determined by evaluating the degree to which empirical calculations of *K* deviated from a null distribution of expected *K* values from 10,000 permutations of trait evolution on the cryonotothenioid tree.

We used PGLS regression under a BM model of evolution to test for correlations between residual eye size and depth occupancy as well as between residual eye size and %B across cryonotothenioid species. Across all tests of correlated evolution, *p* values were adjusted using the false discovery rate corrections method of Benjamini and Hochberg (1995) using the p.adjust R function. We used a Bayesian analysis of macroevolutionary mixtures (BAMM) (Rabosky 2014) to determine if the rate of eye size evolution experienced accelerations or decelerations during the evolutionary history of notothenioids. This analysis allowed us to assess evidence for an elevated rate of eye size evolution during the initial radiation as would be predicted by classical adaptive radiation theory (Gavrilets and Losos 2009; Simpson 1953) versus evidence of more recent eye size diversification following ecological opportunities generated by community recovery from glacial scouring events (Dornburg et al. 2017; Parker et al. 2022). Prior parameters were set using the R package BAMMtools v2.1.1 (Rabosky et al. 2014), and we ran two independent analyses for 50 million generations, sampling every 1000 generations. Convergence between runs was assessed through visual inspection of the log‐likelihoods and effective sampling of the target posterior distribution of parameter values was assessed through quantification of ESS values (ESS > 200). We assessed the sensitivity of our diversification rate analysis to the prior in BAMM (Moore et al. 2016; Rabosky, Mitchell, and Chang 2017) with replicate analyses favoring 0, 1, and 2 rate shifts. We complimented this analysis using the timeSlice algorithm in transformPhylo.ML function within the motmot v.2.1.3 R package (Puttick et al. 2020). Using an approach based on that developed by Slater (2013), we tested if trait diversification has been higher in the last 5 million years.

As shifts in the rate of trait evolution may not correspond to shifts in selective regimes, we assessed evidence for multiple selective regimes associated with eye size or water depth across the cryonotothenioid phylogeny using the ℓ1ou method (Khabbazian et al. 2016). This method incorporates an OU model and a lasso penalty to detect branches in the phylogeny where shifts toward distinct trait optima occur, without prior assumptions about shift locations. By modeling an adaptive landscape that combines random trait change with selection towards specific optima, we are able to discern whether there are detectable signatures of shifts in selective regimes across the phylogeny. Model selection was based on phylogenetic Bayesian Information Criterion (pBIC) scores.

### 2.3 Identification and Comparison of Cryonotothenioid Opsin Sequences

Zebrafish opsin sequences LWS1 (BAC24127.1), LWS2 (BAC24128.1), Rh2‐1 (BAC24129.1), Rh2‐2 (BAC24130.1), Rh2‐3 (BAC24131.1), Rh2‐4 (BAC24132.1), SWS1 (BAC24134.1), SWS2 (BAC24133.1), and Rh1‐1 (NP_571159.1) and Rh1‐2 (BAC21668.1) (Chinen et al. 2003; Hamaoka et al. 2002; Morrow, Lazic, and Chang 2011; Morrow et al. 2017) were used as queries for BLASTp searches restricted to Notothenioidei (taxid: 8205), giant grouper ( *Epinephelus lanceolatus* —taxid: 310571), and wolf eel ( *Anarrhichthys ocellatus* —taxid: 433405). We additionally queried available cryonotothenioid genomes on NCBI and the *Eleginops maclovinus* genome (Chen et al. 2019) available through GIGADB (DOI: 10.5524/102163). Sequences were identified using HMMER (Eddy 2009) with an *e*‐value threshold of 10 × e^−6^. Training profiles for HMMer were built using a fasta alignment of known opsin receptors from NCBI identified above. Candidate opsins were aligned to the reference sequence dataset for manual inspection. All protein sequences were aligned using MAFFT (Katoh 2002) and inspected via Aliview (Larsson 2014). As several available cryonotothenioid genomes were sequenced with low (10×) coverage, instances of fragmented opsin genes within scaffolds were excluded from analysis as meaningful tuning site comparisons would not be possible. Additionally, nonvisual extraocular rhodopsin, (exo‐rh1) sequences, which are similar to Rh1 but expressed in the pineal gland (Chen, Samadi, and Chen 2018; Fujiyabu et al. 2019), were excluded from this study based on their phylogenetic placement.

Opsin protein sequences were imported into Geneious Prime 2021.2 (https://www.geneious.com) and the identity of opsins was assessed using maximum likelihood based phylogenetic inference in IQ‐TREE2 (Minh et al. 2020) conditioned on the best fit model of amino acid substitution with the candidate pool of substitution rates spanning all common amino acid exchange rate matrices (JTT, WAG, etc). The candidate pool of substitution models additionally included protein mixture models such as empirical profile mixture models (Minh et al. 2021; Quang, Gascuel, and Lartillot 2008), as well as parameters to accommodate among‐site rate variation (discrete gamma or free rate model). The best fit model for each alignment was selected using Bayesian information criterion with node support assessed via 1000 ultrafast bootstrap replicates.

## 3 Results

### 3.1 Ecological and Morphological Evolution

**FIGURE 1:**
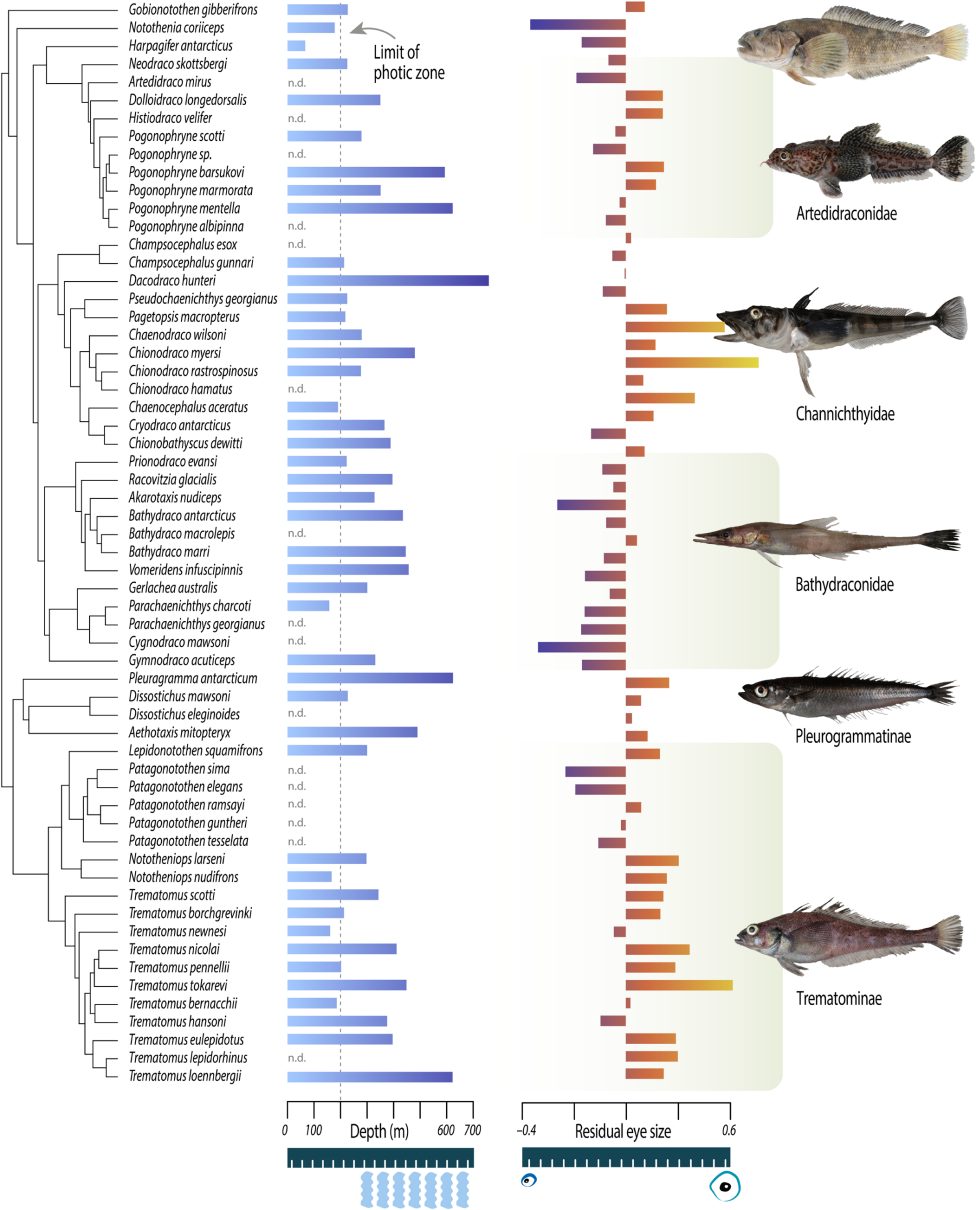
Visualization of variation in mean depth and eye size across the cryonotothenioid phylogeny. Shown on the left panel is a time‐calibrated tree depicting phylogenetic relationships among cryonotothenioid species sampled in our morphological dataset. The middle panel depicts average depth per species, with darker shadings corresponding to deeper depths. The right panel depicts a barplot of eye size (represented as residuals from the regression of eye diameter on SL) measured for our focal cryonotothenioid species with warm colors representing larger eyes relative to head size. Fish images: EP.

In all cases, analysis using HL or SL as a measure of body size yielded equivalent results (Supporting Information), so we focus here on results based on regression of eye size against SL. Analysis of depth data for cryonotothenioids revealed numerous instances of taxa transitioning to depths well beyond the photic zone (Figure 1). Simultaneous visualization of variation in mean depth occupancy and residual eye size corrected for body size across the cryonotothenioid phylogeny reveals that both the deepest‐ and shallowest‐dwelling cryonotothenioid species occupy overlapping extremes of variation in eye size (Figure 1). For example, *Pleurogramma antarcticum* is often encountered deeper than 500 m and possesses large eyes relative to body size, while the similarly depth distributed *Pogonophryne mentella* exhibits small eye size relative to body size (Figure 1).

**FIGURE 2:**
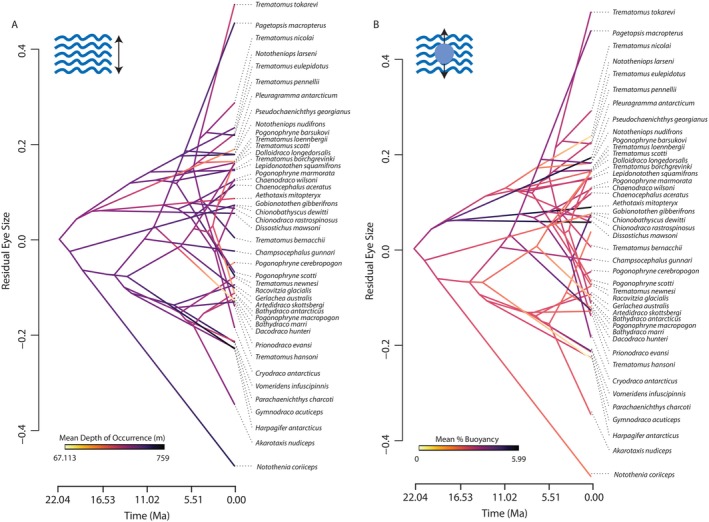
Projection of cryonotothenioid phylogeny in space defined by time and eye size. Time (in millions of years) is on the X axis and eye size variability is on the Y axis. Placement of tree tips along the Y axis corresponds to eye size (represented using the residuals from regression of eye diameter on head length) for each cryonotothenioid species. Ancestral state reconstructions of mean depth of occurrence (panel A) and of mean %B (panel B) have been mapped onto the cryonotothenioid phylogeny to facilitate simultaneous visualization of variation in eye size and variation in water column usage.

Similarly, among cryonotothenioid species with the largest relative eye sizes, we identify examples of both neutrally buoyant pelagic species (e.g., *Pleuragramma antarcticum* ) and less‐buoyant benthic species (e.g., *Nototheniops nudifrons* ) (Figure 2A). Contrasting patterns of eye size evolution relative to either %B (Figure 2A) or depth (Figure 2B) reveals numerous examples of convergence in these habitat traits with divergences in eye size. These contrasts are most pronounced in channichthyids and trematomines (Figure 2; Supporting Information), suggesting an asymmetry in the evolution of eye sizes between clades. In total, these results reveal extreme instances of convergence in eye size and depth occupancy across the evolutionary history of cryonotothenioids (Figure 2), with no evidence for a range of eye sizes statistically associated with specific depth‐niche from phylogenetic ANCOVAs (0.07 < *p* < 0.98).

**FIGURE 3:**
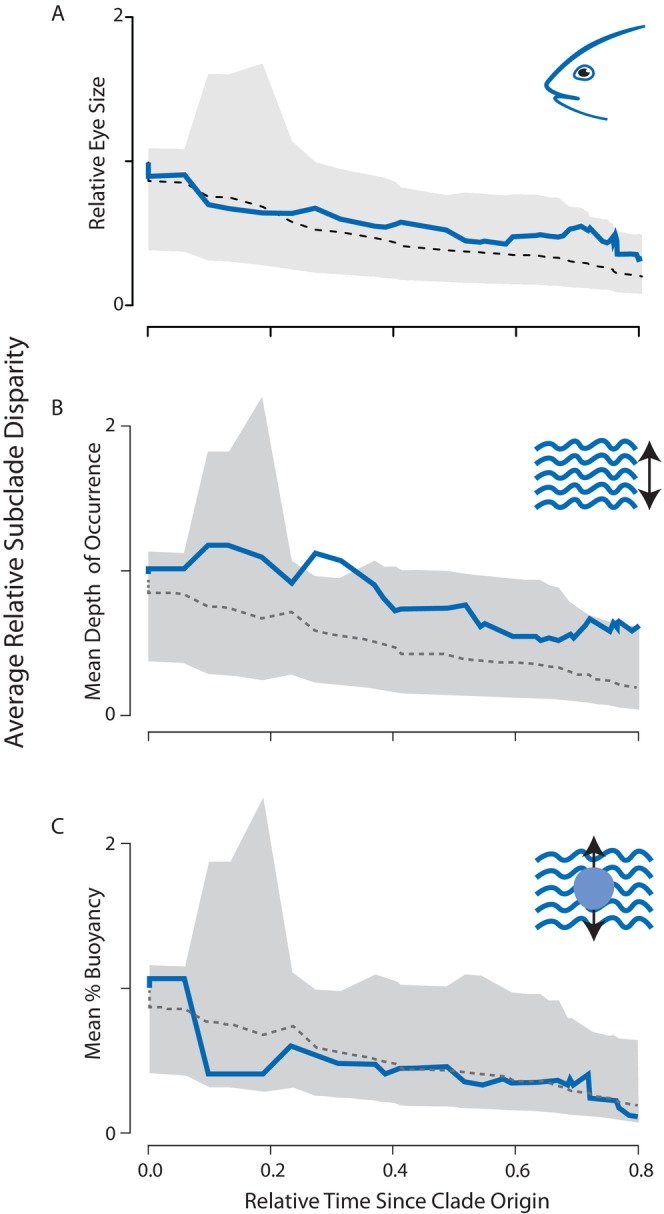
Disparity through time (DTT; Harmon et al. 2003) in eye size and ecology over the course of the cryonotothenioid radiation. Panel A depicts patterns of disparity in residual eye size corrected for head length, panel B reflects disparity in mean depth of occurrence, and panel C depicts disparity in mean % buoyancy. In all plots, the X axis reflects relative time since clade origin (0.0). The Y axis corresponds to average relative subclade disparity in eye size. The solid blue line depicts the empirical estimation of eye size disparity, while the dotted gray line depicts the median trait disparity calculated from 10,000 Brownian motion simulations of trait evolution on the cryonotothenioid phylogeny. The shaded gray region represents the 95% confidence interval (CI) of the Brownian motion simulations.

Analyses of relative subclade disparity in these traits through time (Figure 3) also detected no evidence for an early burst of trait diversification. Instead, residual eye size and %B generally followed the mean expectations of a BM model, with no elevated within clade diversification (i.e., most variation was partitioned among distinct subclades; Figure 3A,C). This lack of within clade disparity in these traits corresponded with the strong phylogenetic signal detected using Pagel's lambda statistic and Blomberg's *K* (Table 1; Supporting Information), suggesting that the rapid divergences in residual eye size variation between closely related taxa (Figure 2) are not a general feature of this radiation. This result is further supported by a visualization of residual eye size variation overall (Figure 2) and the lack of shifts in adaptive regimes detected by the *ℓ1ou* method. Comparison of different models of eye size evolution across notothenioids revealed the OU (AICc = −21.65) and delta (AICc = −21.50923) models to be nearly equally supported (Table 2). As such, evolutionary models lend equivalent support to rapid eye size evolution a bounded continuum, an increase in eye size diversification toward the present, or some combination of the two.

**TABLE 1:** Results of tests of phylogenetic signal in ecomorphological traits.

Traits	Pagel's lambda		Blomberg et al.'s *K*	
	Estimate	*p*	Estimate	*p*
Residual eye size	0.508	0.00038	0.518	0.0007
Mean depth of occurrence	7.3E−05	1	0.43	0.28
Mean % buoyancy	1.04	5.60E−06	0.88	1.0E−04

**TABLE 2:** Comparison of evolutionary model fits to residual eye size.

Model	AICc	AICc weight
Ornstein–Uhlenbeck	−21.65	0.460
Delta	−21.51	0.420
Brownian motion	−18.42	0.090
Evolutionary burst	−16.21	0.029
White noise	−10.66	0.001

In contrast, mean depth of occurrence depicts a signature of higher within than between clade disparity (Figure 3B), corresponding to divergences between closely related species and convergences in eye size and depth niche between distantly related species. Relative to the phylogeny, the most extreme divergence occurs in *Cryodraco*, which is the only branch supported as being under an alternative selective regime by the *ℓ1ou* method, complimenting the finding of high within clade disparity of depth occupancy. Results of our PGLS regression analysis revealed no significant evidence that eye size is correlated with mean depth of occurrence, minimum reported depth, or maximum reported depth across cryonotothenioid (*p* > 0.05 for all regressions; Table 3) regardless of the underlying model of trait evolution. We further find no significant evidence that shifts in eye size are associated with predictable shifts in %B (*p* > 0.05; Table 3).

**TABLE 3:** Results from PGLS regressions of residual eye size (based on SL) on depth and buoyancy for all notothenioid species.

Predictor variable	Parameter estimate (β) ± SE	*t*	*p* ^a^
Mean depth of occurrence	−0.00012 ± 0.00017	0.69	0.48
Minimum reported depth	−0.00007 ± 0.0001	0.72	0.48
Maximum reported depth	−0.00009 ± 0.00004	2.22	0.12
Mean % Buoyancy	−0.02067 ± 0.0270	−0.76	0.48

^a^ *p* values were adjusted to correct for multiple comparisons using the false discovery rate correction method of Benjamini and Hochberg (1995).

### 3.2 The Tempo of Eye Size Diversification

**FIGURE 4:**
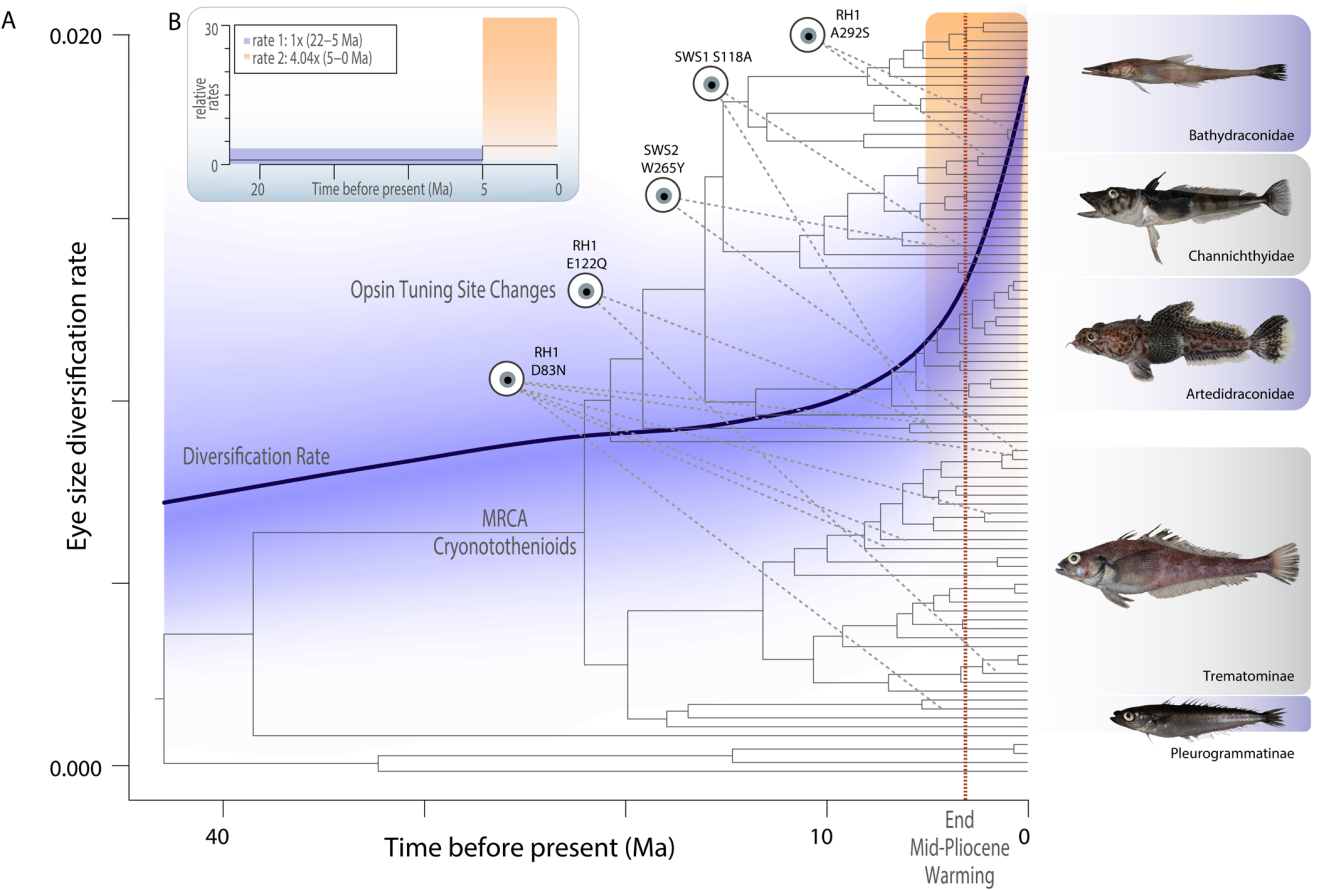
Punctuated elevation in the diversification of eye size well after the onset of the cryonotothenioid adaptive radiation. (A) The mean rate of eye size diversification (solid line) and confidence interval (blue shading) based on BAMM overlaid on the time calibrated phylogeny of cryonotothenioids and key opsin substitutions discussed in the text (dotted purple lines), revealing a notable increase in eye size diversification coincident with the end of the mid‐Pliocene warming (orange line) that occurred after the initial Antarctic radiation (gray text; Daane et al. 2020; Near et al. 2012). (B) Relative rates of residual eye size diversification across 5 million year time slices estimated by motmot. Tall light shaded box in B corresponds to the light shaded box in A. Photos: EP.

A Bayesian quantification of the rate of eye size diversification reveals a rapid and recent acceleration of eye size diversification through time (Figure 4). Following the onset of the initial adaptive radiation believed to have occurred at the most recent common ancestor of cryonotothenioids, the rate of eye size diversification remains relatively unchanged throughout the initial emergence of the major cryonotothenoid subclades. However, our BAMM analysis supports a marked shift in the rate of eye size diversification beginning in the last 5 million years, reflecting the sometimes dramatic divergences in relative eye size between closely related taxa (Figure 1). This uptick in diversification rate is also supported by a motmot timeslice analysis, that quantifies a four‐fold increase in the rate of eye size diversification during the time interval identified by BAMM (Figure 4).

### 3.3 The Evolution of Cryonotothenioid Opsin Tuning Sites

**FIGURE 5:**
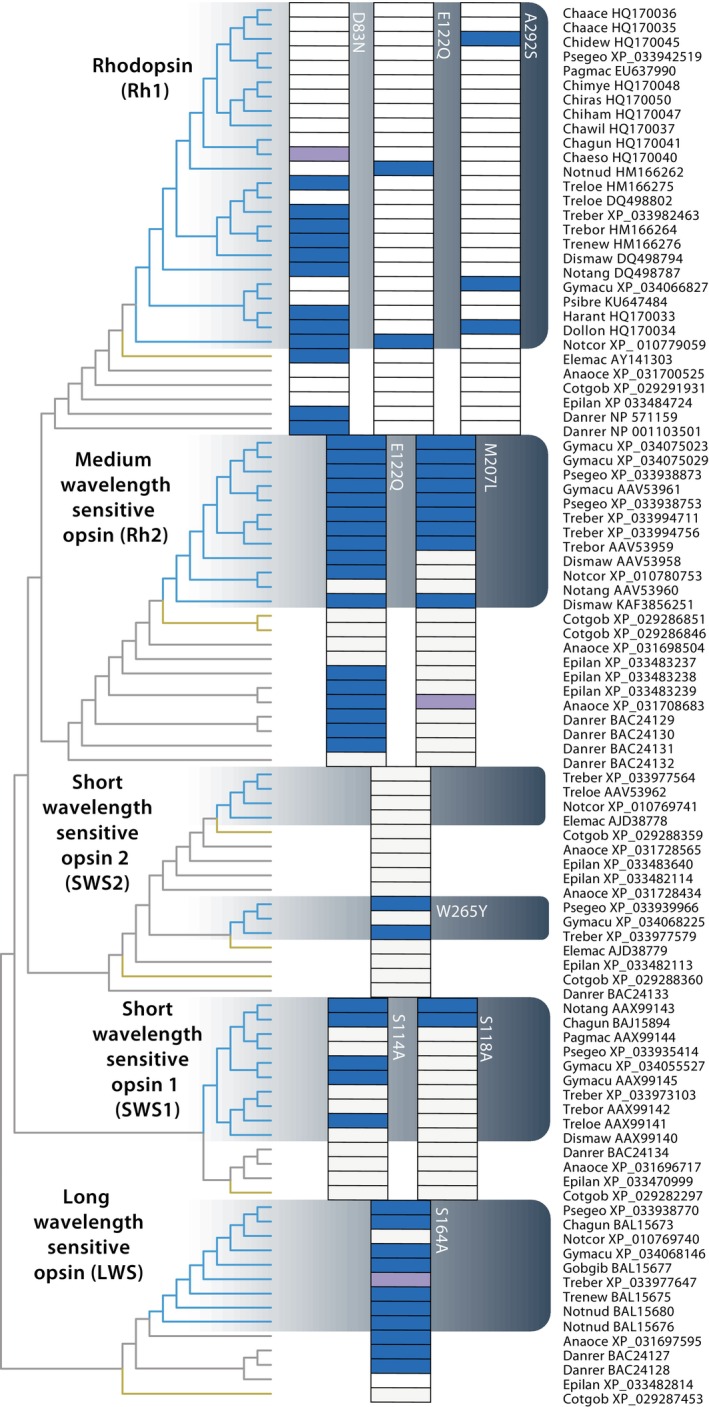
Evolution of opsin tuning site replacements in cryonotothenioids. Maximum likelihood topology of opsin sequences (left) with tuning site replacements indicated in the shaded grids. Clades identity is indicated on the phylogeny and cryonotothenoids are indicated by the gray gradient box and blue shading on the phylogeny. Blue boxes indicate the presence of a substitution and purple boxes indicate alternate substitutions (see supplemental materials). Other nototheniods are indicated on the phylogeny in yellow, other teleost outgroups in gray. Text near the shaded boxes indicates AA replacement (i.e., W265Y). Names on the right provide sequence accession numbers and genus/species codes. Genus, species, and common names are provided in Table S2.

We identified opsin sequences from 25 notothenioids including 23 cryonotothenioids (Supporting Information) and confirmed their classification as Rh1, Rh2, SWS2, SWS1, or LWS through phylogenetic analyses (Figure 5). Our results reveal a complex history of tuning site substitutions with numerous independent shifts toward lower frequency light wavelengths within cryonotothenioids. By comparing cryonotothenioid opsin sequences to other notothenioids along with perciforms and teleost outgroups, we identified a number of opsin changes that may influence their maximum wavelength of absorbed light *λ*_max_ (Figure 5; Supporting Information).

The four tuning sites of teleost Rh1 (when mutated) can significantly shift the *λ*_max_ are D83 E122, F261 and A292 (Bowmaker 2008; Lin et al. 2017; Yokoyama 2008), and we observed multiple instances of D83N (−3 nm), and sporadic occurrences of E122Q (−13 nm) and A292S (−2 nm), as well as combined D83N and A292S substitutions (−14–17 nm) in cryonotothenioid Rh1 sequences (Figure 5; Supporting Information). We identified tuning site changes, E122Q and M207L, in the majority of cryonotothenioid Rh2 sequences, with some lineage specific reversals in *Notothenia* and *Dissostichus* (Figure 1; Figure S2 and Table S4). These substitutions could decrease *λ*_max_ of Rh2 by up to 17 and 10 nm (E122Q and M207L, respectively) (Takenaka and Yokoyama 2007; Yokoyama et al. 1999; Yokoyama and Jia 2020). In contrast to this signature of amino acid replacements during the evolution of Rh2, the W265Y replacement in one copy of SWS2 revealed a convergence in a nearly 30 nm shift toward smaller light wavelengths (Yokoyama, Takenaka, and Blow 2007) that occurred independently in two distantly related species ( *Pseudochaenichthys georgianus* and *Trematomus bernacchii* (Figure 5; Supporting Information)). Similar independent convergences also occurred in SWS1 replacements S114A and S118A (Figure 5; Supporting Information). Finally, we identified two distinct changes in a single tuning site: S164A and S164P cryonotothenioid LWS sequences, the latter representing a unique substitution in *Trematomus bernacchi* (Figure 5; Supporting Information). Collectively, almost all of these changes in cryonotothenioid opsins are predicted to result in decreases in *λ*_max_ with shifts toward shorter wavelengths (Supporting Information).

Contrasting these shifts in eye size and depth occupancy with tuning site replacements further revealed the lack of a consistent ecomorphological signal. For example, *Trematomus loennbergii* and *Champsocephalus gunnari* have converged on the SWS1 replacement S114A (Figures 4 and 5). However, *Trematomus loennbergii* exhibits a substantial enlargement of eyes relative to head/body size and occupies depths of over 600 m (Figure 1; Supporting Information), while the distantly related *Champsocephalus gunnari* commonly occupies depths less than 300 m and exhibits no dramatic eye size enlargement. Similarly, *Dolloidraco*, *Chionobathyscus*, and *Gymnodraco* all converge on an A292S replacement in Rh1 following transitions to deeper water (Figures 1, 4, and 5). *Dolloidraco* and *Chionobathyscus* exhibit no substantial eye enlargement or reduction, while *Gymnodraco* ranks among the cryonotothenioids with the smallest eyes relative to body/head size (Figure 1; Supporting Information).

## 4 Discussion

For a closely related group of species, evolutionary radiation across the water column may be expected to require a correlation between the diversification of depth niche, eye size, and the molecular basis of light detection (Huber et al. 1997). However, our results strongly suggest such an expectation does not characterize the cryonotothenioid adaptive radiation. Instead, we reveal a complex history of trait convergences and divergences that collectively suggest evolutionary changes in opsin tuning sites, eye size, depth niche, and buoyancy may be largely decoupled in this radiation. Our analysis reveals that convergences in depth result in no clear association with eye size or buoyancy. Lineages occupying similar depths give rise to extremes in eye size and often exhibit different levels of buoyancy. Eye size divergence is often high among closely related taxa, and our results further reveal that the rate of eye size diversification in cryonotothenioids accelerated at a time coincident with recovery from the end of the mid‐Pliocene warming event. We further find a consistent signature of shifts in tuning sites reflecting shifts towards lower wavelengths of light that represent repeated instances of independent tuning site changes among distantly related species that are not necessarily associated with convergences in depth or eye size. Collectively, our results reveal that the diversification of these key components of the visual system do not conform to expectations of the classic adaptive radiation model.

### 4.1 Evolving to Gaze Into the Abyss

Changes in water column usage are a major axis of diversification in ray‐finned fishes (Brownstein, Dornburg, and Near 2024; Daane et al. 2019; Eastman and McCune 2000; Parker et al. 2022), and the stratification of cryonotothenioids across the water column of the Southern Ocean is a hallmark of their evolutionary success (Daane et al. 2019; Eastman and McCune 2000; Parker et al. 2022). However, we found no strong evidence for coordinated evolutionary changes between eye size, depth niche, or buoyancy. Instead, we found repeated convergences in depth niches and eye size diversity between lineages, suggesting that these traits are highly labile. In particular, relative eye size changes appear most pronounced within Channichthyidae and Trematominae, two lineages previously identified as nested adaptive radiations (Near et al. 2012; Parker et al. 2022). In these clades, our analyses reveal multiple instances of closely related taxa sharply diverging in eye size, resulting in a high degree of eye size convergence between distantly related taxa.

Our quantification of the rate of eye size evolution (Figure 4) likely reflects the recovery of lineages following ice scour events that decimated near‐shore ecological communities (Dornburg et al. 2017; Parker et al. 2022; Thatje, Hillenbrand, and Larter 2005; Thatje et al. 2008). As lineages colonize recovering communities, stratification of the vertical water column presents a mechanism to mitigate resource conflict and explore additional ecological opportunities (La Mesa, Eastman, and Vacchi, 2004). The frequency of these transitions may derive from a predisposition to dim‐light vision in cryonotothenioids, which could explain the lack of evidence for any shifts in selective regimes surrounding eye size. The depth ranges occupied by cryonotothenioids tend to be deeper than most “near‐shore” fauna given the depression of continental shelf, and polar latitudes experience extreme seasonal fluctuations in sunlight along with shifts in light attenuation due to ice coverage and phytoplankton biomass (Tilzer et al. 1994). We suggest that such conditions have likely primed cryonotothenioids to repeatedly converge in depth occupancy patterns across hundreds of meters in short evolutionary timescales.

Larger eyes are associated with an increase in photoreceptors that can collect higher amounts of sensory information (Iglesias et al. 2018). While such increases could be expected to aid lineages in prey detection, we found that the evolution of eye size and depth niche are not correlated in cryonotothenioids. We suggest that this lack of correlation between eye size and depth niche may reflect tradeoffs in the neurological investment between chemoperception and vision. Across vertebrates, eye size has repeatedly been shown to be linked with aspects of brain architecture, especially those associated with vision (Burton 2008; Corral‐López et al. 2017; Howell et al. 2021). Many predatory teleosts that hunt in dim‐light conditions have reduced eye sizes and correspondingly reduced optic tecta, instead relying on olfaction and concomitant increases in the size of the brain's olfactory bulb for prey detection (Edmunds, McCann, and Laberge 2016; Iglesias et al. 2018; Yamamoto 2017). In contrast, fishes that constitute the prey of such lineages foraging in open environments often rely on vision to detect incoming motion and ambush predators (Guthrie 1990; Kotrschal et al. 2017). As some predatory cryonotothenioids are themselves primary prey items for marine mammals and birds (Casaux, Bertolin, and Carlini 2011; Eastman 1985; La Mesa, Eastman, and Vacchi 2004), predation pressure could be a major force shaping the evolution of cryonotothenioids eye size and associated investment in corresponding brain regions. Such a hypothesis would be in line with growing evidence that predation pressure can impact the evolution of the brain within (White and Brown 2015) and between species (Kotrschal et al. 2017). Future studies contrasting neural investment patterns with ecological data across the phylogeny of cryonotothenioids are needed to assess the degree to which eye size and brain evolution have been impacted by predation pressure.

An additional key trait to the cryonotothenioid radiation in the water column is their ability to modulate buoyancy. As all notothenioids lack a swim bladder, changes in skeletal ossification permit cryonotothenioids to utilize deeper or shallower water column niches (Daane et al. 2019; Daane and William Detrich 2021). However, we find a surprising lack of correlation between buoyancy and depth of occurrence. This lack of correlation is likely explained by evolutionary transitions along the benthic‐pelagic axis of the water column. Benthic species will naturally be more negatively buoyant and co‐occur with pelagic species across the depth range of cryonotothenioids. Unfortunately, understanding the relationship between traits in this study and this aspect of the water column niche is complicated by uncertainty in classification of cryonotothenioid species into benthic or pelagic categories. Some species, such as *Pleuragramma antarcticus*, *Dissostichus mawsoni* , *D. eleginoides* , and *Aethotaxis mitopteryx* , are unquestionably pelagic (Eastman 2020; Eastman and DeVries 1982; Near et al. 2003, 2007), and others, such as species of spiny plunderfish *Harpagifer*, can almost certainly be considered benthic (Eastman 2020). On the other hand, several cryonotothenioids have been observed to opportunistically forage outside of their “expected” water column niche, including benthic species opportunistically capturing pelagic prey (Daniels 1982). However, such data are extremely limited. Continued ecological investigations focused on how cryonotothenioids are utilizing their habitat are needed to place our analyses into a more refined ecological context, as our ability to accurately contrast patterns of eye size or opsin evolution relative to these aspects of water column niches, is currently limited. Fortunately, continual advances in imaging technology hold the promise of overcoming traditional logistical challenges of observing cryonotothenioids in the Antarctic (Jones and Near 2012; La Mesa et al. 2021; Purser et al. 2022) and providing these critically needed insights into the ecology of this adaptive radiation in the near future.

### 4.2 The Mosaic Evolution of Cryonotothenioid Opsins

Closely related cryonotothenioid species often diverge in their water column niches by hundreds of meters, subjecting them to new ecological opportunities and photic conditions (Parker et al. 2022). While it has been established that changes in opsin tuning sites can result in increases or decreases in the maximum wavelength of absorbed light (*λ*_max_) (Lin et al. 2017; Bowmaker 2008; Yokoyama 2008), changes in opsin sites are often not consistent relative to depth niche between cryonotothenioid species. Likewise, there is no evidence that changes in eye size correspond to consistent changes in tuning sites. Instead, cryonotothenioids have maintained some tuning sites that likely arose prior to or coincident with their early radiation as well as with numerous substitutions that arose throughout the radiation in various lineages. For example, based on empirical research in other fishes, it is expected that the observed E122Q substitution within Rh2 of notothenioids would decrease *λ*_max_ by 13–17 nm (Takenaka and Yokoyama 2007). The E122Q substitution is found in nearly all cryonotothenioid Rh2 sequences we investigated (with the exception of a reversal in *Notothenia angustata* ). This substitution is also found in a wide range of teleost lineages including other Perciformes (Lin et al. 2017), indicating that the E122 in a genus of thornfishes, *Cottoperca*, could reflect a reversal. The M207L mutation in Rh2 is also pervasive across most surveyed cryonotothenioids, and predicted to decrease *λ*_max_ by 6 nm (Yokoyama et al. 1999). As the M207L mutation has been found in only a few teleost lineages (e.g., *Fundulus*, *Xiphophorus* and *Poecilia*) (Lin et al. 2017) but not in other Perciformes included in this study (Figure S2), this suggests the M207L mutation may be a unique feature of cryonotothenioids.

In contrast to these widespread substitutions, we observe the combination of D83N with A292S in the Rh1 of *Chionobathyscus dewitti* and *Gymnodraco acuticeps* that likely reflect a *λ*_max_ decrease of ~13–17 nm (Yokoyama et al. 2008). The A292S change has previously been identified as an adaption for deeper water in cichlids (Ricci et al. 2022; Sugawara et al. 2005), and the instance of this substitution in *Chionobathyscus dewitti* and *Gymnodraco acuticeps* likely reflects an evolutionary convergence in these deeper dwelling taxa. However, these mutations are not uniform in all deep dwelling taxa occurring at similar or deeper depths. We also observed an E122Q mutation in Rh1 in the distantly related *Nototheniops nudifrons* and *Notothenia coriiceps* that likely reflects a similar *λ*_max_ decrease in ~13 nm (Yokoyama et al. 2008). In a recent survey of ray‐finned fish opsins, the E122Q mutation was found in *Notothenia coriiceps* (the only notothenioid in their report) and three other teleosts: Pacific bluefin tuna ( *Thunnus orientalis* ), yellowhead catfish (*Tachysurus fulvidraco*), and red piranha ( *Pygocentrus nattereri* ) (Lin et al. 2017). Similarly, the double mutation of D83N/A292S was only observed in a few other teleost lineages: red piranha ( *Pygocentrus nattereri* ), blind cave fish ( *Astyanax mexicanus* ), the eel species *Anguilla anguilla* and *A. japonica* , and ballan wrasse ( *Labrus bergylta* ) (Lin et al. 2017). The rarity of these substitutions across all ray‐finned fish lineage compared with the frequency of our observations in cryonotothenioids suggests these are likely more widespread across other cryonotothenioids and will be an exciting area of future functional and ecological studies.

To date, no report has identified all opsins from an individual notothenioid species and little information has been reported about their tuning sites. While we confirm and expand the scope of changes previously identified in a limited number of notothenioids, such as those in SWS1 (S114A; S118A), Rh2 (E122Q; M207L) (Pointer et al. 2005), LWS (S164A) (Miyazaki and Iwami 2012), and Rh1 (E122Q) (Lin et al. 2017), we also identified an undescribed notothenioid substitution that exceeds the spectral shift of any previously identified substitution. We found a W265Y substitution in SWS2 sequences in the distantly related *Pseudochaenichthys georgianus* and *Trematomus bernacchii* that is predicted to lead to a significant decrease in *λ*_max_ by 29 nm (Yokoyama, Takenaka, and Blow 2007). This substitution has been observed in only a few teleost lineages (e.g., killifish), where there are two copies of SWS2, in which SWS2‐A retains W265 and blue sensitivity, and SWS2‐B provides violet sensitivity through a Y265 substitution (Lin et al. 2017; Yokoyama, Takenaka, and Blow 2007). Our analyses suggest that *Trematomus bernacchii* encodes SWS2‐A and SWS2‐B (Figure 4), and more sequencing is needed from other notothenioids to determine whether all notothenioids encode both SWS2 opsins. If W265Y is indeed more pervasive across cryonotothenioids, this would suggest a substantial shift towards shorter blue/UV light wavelengths and further underscores a key result of our study: Functional changes in cryonotothenioid opsins represent an evolutionary mosaic shifted toward shorter light wavelengths. How the frequency and nature of these molecular changes compare to other teleost clades occupying polar or dim light environments represents an intriguing topic for future research.

## 5 Conclusion

It has become clear over the last decade that the adaptive radiation of cryonotothenioid is far more complex than previously hypothesized. Rather than merely representing a clade that radiated at the onset of polar conditions in the Antarctic over 30 million years ago (Eastman and McCune 2000; Rutschmann et al. 2011), this radiation is comprised of nested radiations that have arisen in response to recent warming events in the Plio/Pleistocene and ongoing cycles of ice scouring across the continental shelf (Bista et al. 2023; Dornburg et al. 2017; Near et al. 2012; Parker et al. 2022). Our results are consistent with this emerging view, demonstrating an acceleration of eye size evolution following the mid‐Pliocene warming event, and high levels of ecological and morphological convergence between distantly related species. We further reveal that the diversification of cryonotothenioid opsins reflects a complex evolutionary mosaic that includes both molecular substitutions in tuning sites that occurred early in the group's history as well as recent reversals and novel substitutions. Collectively, patterns of depth usage, tuning site substitutions, or eye sizes are heterogeneous even among close evolutionary relatives with convergences in taxa that are otherwise highly divergent in other aspects of life history. This potential decoupling of ecological, morphological, and genotypic traits raises the possibility that the relationship between ecological functions and visual acuity may represent a many‐to‐one mapping of forms to function, similar to that often observed in studies of feeding performance and morphology (Alfaro, Bolnick, and Wainwright 2005; Wainwright et al. 2005). As changes in eye size are often correlated with changes in neural investment (Howell et al. 2021; Iglesias et al. 2018), future comparative considerations of transitions in depth and the compositional diversity of cryonotothenioid brains offers an exciting research frontier that can illuminate the pathways and tradeoffs that underlie neural diversification in this adaptive radiation. Given the environmental threats facing the Southern Ocean (Patarnello et al. 2011; Vacchi, Pisano, and Ghigliotti 2017), such studies are also of high conservation importance for understanding the impact of warming surface waters on lineages predicted to alter their depth niches in response.

## Author Contributions

**Ella B. Yoder:** data curation (equal), formal analysis (equal), investigation (equal), methodology (equal), visualization (equal), writing – original draft (equal), writing – review and editing (equal). **Elyse Parker:** conceptualization (equal), data curation (equal), formal analysis (equal), investigation (equal), methodology (equal), validation (equal), writing – original draft (equal), writing – review and editing (equal). **Bruno Frédérich:** data curation (equal), formal analysis (equal), methodology (equal), supervision (equal), validation (equal), writing – review and editing (equal). **Alexandra Tew:** data curation (equal), investigation (equal), validation (equal), visualization (equal). **Christopher D. Jones:** conceptualization (equal), resources (equal), supervision (equal), writing – review and editing (equal). **Alex Dornburg:** conceptualization (equal), formal analysis (equal), methodology (equal), project administration (equal), supervision (equal), visualization (equal), writing – original draft (equal), writing – review and editing (equal).

## Ethics Statement

The authors have nothing to report.

## Consent

The authors have nothing to report.

## Conflicts of Interest

The authors declare no conflicts of interest.

## Supporting information


Data S1.

## Data Availability

All data generated or analyzed during this study are included in this published article (and its Supporting Information files). Code, data, and trees to replicate analyses are available on Zenodo (DOI: 10.5281/zenodo.7116757): https://zenodo.org/records/14537357.
