# Supplementary material for: Multiple Pathways of Visual Adaptations for Water Column Usage in an Antarctic Adaptive Radiation
Source: Ecol Evol. 2025 Mar 9;15(3):e70867. doi: 10.1002/ece3.70867 (PMC11890982; doi:10.1002/ece3.70867)
Supplement: Supplementary file 1 — Data S1. [file ECE3-15-e70867-s001.pdf]

- Supplementary Tables and Figures -

Multiple pathways of visual adaptations for water column usage in an Antarctic adaptive radiation

Table of Contents

Table S1. List of specimens used for morphological analyses 2

Table S2. Genus, species, abbreviations, and common names in Figures 4 and S1-S5 6

Table S3. Opsin sequence identifiers included in Figures 4 and S1-S5 7

Table S4. Changes in major tuning sites and their predicted λmax. 8

Table S5. Results from PGLS regressions of residual eye size (based on HL) on depth and buoyancy for all notothenioid species 9

Table S6. Results of tests of phylogenetic signal in residual eye size relative to head size 9

Figure S1. Notothenioid Rh1 sequences and tuning sites 11

Figure S2. Notothenioid Rh2 sequences and tuning sites. 13

Figure S3. Notothenioid SWS2 sequences and tuning sites. 15

Figure S4. Notothenioid SWS1 sequences and tuning sites. 17

Figure S5. Notothenioid LWS sequences and tuning sites. 19

Figure S6. Phylomorphospace plot of depth versus residual eye size. 20

Figure S7. Phylogenetic signal in ecomorphological traits. 21

Figure S8. Correlation between morphological traits. 22

Figure S9. Visualization of morphological measurements. 23

Figure S10. Visualization of variation in mean depth and eye size across the notothenioid phylogeny 24

Figure S11. Projection of notothenioid phylogeny in space defined by time and residual variation in eye size 25

Figure S12. Disparity through time (DTT; Harmon et al. 2003) over the course of the notothenioid radiation. 26

References 27

Table S1. List of specimens used for morphological analyses.

Clade	Genus species	Museum Voucher[1]
Bathydraconidae	*Akarotaxis nudiceps*	YPM ICH 24241
Bathydraconidae	*Akarotaxis nudiceps*	YPM ICH 20210
Bathydraconidae	*Akarotaxis nudiceps*	YPM ICH 20037
Bathydraconidae	*Bathydraco antarcticus*	NMNZ P043669
Bathydraconidae	*Bathydraco antarcticus*	NMNZ P043670
Bathydraconidae	*Bathydraco macrolepis*	NMNZ P042270
Bathydraconidae	*Bathydraco macrolepis*	NMNZ P043387
Bathydraconidae	*Bathydraco marri*	NMNZ P040098
Bathydraconidae	*Bathydraco marri*	YPM ICH 22590
Bathydraconidae	*Bathydraco marri*	YPM ICH 22477
Bathydraconidae	*Cygnodraco mawsoni*	NMNZ P040018
Bathydraconidae	*Gerlachea australis*	YPM ICH 16476
Bathydraconidae	*Gerlachea australis*	YPM ICH 16642
Bathydraconidae	*Gerlachea australis*	NMNZ P043338
Bathydraconidae	*Gymnodraco acuticeps*	YPM ICH 16635
Bathydraconidae	*Gymnodraco acuticeps*	YPM ICH 22376
Bathydraconidae	*Gymnodraco acuticeps*	NMNZ P043639
Bathydraconidae	*Parachaenichthys charcoti*	YPM ICH 22649
Bathydraconidae	*Parachaenichthys charcoti*	YPM ICH 22649
Bathydraconidae	*Parachaenichthys charcoti*	YPM ICH 22649
Bathydraconidae	*Parachaenichthys georgianus*	YPM ICH 21533
Bathydraconidae	*Parachaenichthys georgianus*	YPM ICH 21533
Bathydraconidae	*Parachaenichthys georgianus*	YPM ICH 21533
Bathydraconidae	*Prionodraco evansii*	YPM ICH 22536
Bathydraconidae	*Prionodraco evansii*	YPM ICH 22689
Bathydraconidae	*Prionodraco evansii*	YPM ICH 24283
Bathydraconidae	*Racovitzia glacialis*	YPM ICH 16660
Bathydraconidae	*Racovitzia glacialis*	YPM ICH 20044
Bathydraconidae	*Racovitzia glacialis*	YPM ICH 22370
Bathydraconidae	*Vomeridens infuscipinnis*	YPM ICH 22391
Bathydraconidae	*Vomeridens infuscipinnis*	YPM ICH 22391
Bathydraconidae	*Vomeridens infuscipinnis*	YPM ICH 22391
Channichthyidae	*Chaenocephalus aceratus*	YPM ICH 24284
Channichthyidae	*Chaenocephalus aceratus*	YPM ICH 24284
Channichthyidae	*Chaenocephalus aceratus*	YPM ICH 24284
Channichthyidae	*Chaenodraco wilsoni*	YPM ICH 24286
Channichthyidae	*Chaenodraco wilsoni*	YPM ICH 21474
Channichthyidae	*Chaenodraco wilsoni*	YPM ICH 16641

[1] Institutional museum collection acronyms follow Sabaj (2016).

Clade	Genus species	Museum Voucher[1]
Channichthyidae	*Champsocephalus esox*	no tag
Channichthyidae	*Champsocephalus gunnari*	YPM ICH 21535
Channichthyidae	*Champsocephalus gunnari*	YPM ICH 21536
Channichthyidae	*Champsocephalus gunnari*	YPM ICH 20242
Channichthyidae	*Chionobathyscus dewitti*	YPM ICH 20010
Channichthyidae	*Chionobathyscus dewitti*	YPM ICH 20011
Channichthyidae	*Chionobathyscus dewitti*	YPM ICH 24123
Channichthyidae	*Chionodraco hamatus*	no tag
Channichthyidae	*Chionodraco hamatus*	no tag
Channichthyidae	*Chionodraco myersi*	YPM ICH 22339
Channichthyidae	*Chionodraco myersi*	YPM ICH 16533
Channichthyidae	*Chionodraco rastrospinosus*	YPM ICH 22341
Channichthyidae	*Chionodraco rastrospinosus*	YPM ICH 22341
Channichthyidae	*Chionodraco rastrospinosus*	YPM ICH 20052
Channichthyidae	*Cryodraco antarcticus*	YPM ICH 22605
Channichthyidae	*Cryodraco antarcticus*	YPM ICH 16521
Channichthyidae	*Cryodraco antarcticus*	YPM ICH 22492
Channichthyidae	*Dacodraco hunteri*	YPM ICH 22338
Channichthyidae	*Dacodraco hunteri*	YPM ICH 16569
Channichthyidae	*Pagetopsis macropterus*	YPM ICH 22335
Channichthyidae	*Pagetopsis macropterus*	YPM ICH 16510
Channichthyidae	*Pagetopsis macropterus*	YPM ICH 20477
Channichthyidae	*Pseudochaenichthys georgianus*	YPM ICH 22601
Channichthyidae	*Pseudochaenichthys georgianus*	YPM ICH 22524
Channichthyidae	*Pseudochaenichthys georgianus*	YPM ICH 16637
Harpagiferidae	*Artedidraco mirus*	YPM ICH 23278
Harpagiferidae	*Artedidraco mirus*	YPM ICH 23278
Harpagiferidae	*Artedidraco skottsbergi*	NMNZ P043365
Harpagiferidae	*Artedidraco skottsbergi*	NMNZ P040034
Harpagiferidae	*Artedidraco skottsbergi*	YPM ICH 22372
Harpagiferidae	*Dolloidraco longedorsalis*	YPM ICH 20033
Harpagiferidae	*Dolloidraco longedorsalis*	YPM ICH 20038
Harpagiferidae	*Dolloidraco longedorsalis*	YPM ICH 20030
Harpagiferidae	*Harpagifer antarcticus*	YPM ICH 20810
Harpagiferidae	*Harpagifer antarcticus*	YPM ICH 16630
Harpagiferidae	*Harpagifer antarcticus*	YPM ICH 16630
Harpagiferidae	*Histiodraco velifer*	NMNZ P043412
Harpagiferidae	*Pogonophryne albipinna*	NMNZ P042633
Harpagiferidae	*Pogonophryne barsukovi*	NMNZ P043539
Harpagiferidae	*Pogonophryne barsukovi*	YPM ICH 16518
Harpagiferidae	*Pogonophryne barsukovi*	YPM ICH 16518
Harpagiferidae	*Pogonophryne marmorata*	YPM ICH 16550

Clade	Genus species	Museum Voucher[1]
Harpagiferidae	*Pogonophryne marmorata*	YPM ICH 20496
Harpagiferidae	*Pogonophryne marmorata*	YPM ICH 16550
Harpagiferidae	*Pogonophryne mentella*	NMNZ P042618
Harpagiferidae	*Pogonophryne mentella*	YPM ICH 20495
Harpagiferidae	*Pogonophryne mentella*	YPM ICH 22565
Harpagiferidae	*Pogonophryne scotti*	NMNZ P045673
Harpagiferidae	*Pogonophryne scotti*	NMNZ P043419
Harpagiferidae	*Pogonophryne scotti*	NMNZ P043370
Harpagiferidae	*Pogonophryne* sp. nov.	NMNZ P042640
Nototheniidae	*Aethotaxis mitopteryx*	NMNZ P043384
Nototheniidae	*Aethotaxis mitopteryx*	NMNZ P043377
Nototheniidae	*Aethotaxis mitopteryx*	YPM ICH 22552
Nototheniidae	*Dissostichus eleginoides*	NMNZ P037795
Nototheniidae	*Dissostichus eleginoides*	YPM ICH 17045
Nototheniidae	*Dissostichus mawsoni*	NMNZ P042631
Nototheniidae	*Dissostichus mawsoni*	YPM ICH 20787
Nototheniidae	*Dissostichus mawsoni*	YPM ICH 20787
Nototheniidae	*Gobionotothen gibberifrons*	YPM ICH 16629
Nototheniidae	*Gobionotothen gibberifrons*	YPM ICH 20779
Nototheniidae	*Gobionotothen gibberifrons*	YPM ICH 20724
Nototheniidae	*Lepidonotothen squamifrons*	YPM ICH 22548
Nototheniidae	*Lepidonotothen squamifrons*	YPM ICH 20490
Nototheniidae	*Lepidonotothen squamifrons*	YPM ICH 20490
Nototheniidae	*Notothenia coriiceps*	NMNZ P040097
Nototheniidae	*Notothenia coriiceps*	NMNZ P040096
Nototheniidae	*Notothenia coriiceps*	NMNZ P040096
Nototheniidae	*Nototheniops larseni*	YPM ICH 24132
Nototheniidae	*Nototheniops larseni*	YPM ICH 24132
Nototheniidae	*Nototheniops larseni*	YPM ICH 24132
Nototheniidae	*Nototheniops nudifrons*	YPM ICH 21496
Nototheniidae	*Nototheniops nudifrons*	YPM ICH 21496
Nototheniidae	*Nototheniops nudifrons*	YPM ICH 21496
Nototheniidae	*Patagonotothen elegans*	YPM ICH 21588
Nototheniidae	*Patagonotothen elegans*	YPM ICH 20076
Nototheniidae	*Patagonotothen elegans*	YPM ICH 20076
Nototheniidae	*Patagonotothen guntheri*	YPM ICH 20072
Nototheniidae	*Patagonotothen guntheri*	YPM ICH 20077
Nototheniidae	*Patagonotothen guntheri*	YPM ICH 20077
Nototheniidae	*Patagonotothen ramsayi*	YPM ICH 20124
Nototheniidae	*Patagonotothen ramsayi*	YPM ICH 20124
Nototheniidae	*Patagonotothen ramsayi*	YPM ICH 20124
Nototheniidae	*Patagonotothen sima*	YPM ICH 20125

Clade	Genus species	Museum Voucher[1]
Nototheniidae	*Patagonotothen sima*	YPM ICH 20125
Nototheniidae	*Patagonotothen sima*	YPM ICH 20125
Nototheniidae	*Patagonotothen tessellata*	YPM ICH 20071
Nototheniidae	*Patagonotothen tessellata*	YPM ICH 20071
Nototheniidae	*Patagonotothen tessellata*	YPM ICH 20071
Nototheniidae	*Pleuragramma antarcticum*	NMNZ P043380
Nototheniidae	*Pleuragramma antarcticum*	YPM ICH 16405
Nototheniidae	*Pleuragramma antarcticum*	YPM ICH 16405
Nototheniidae	*Trematomus bernacchii*	YPM ICH 22595
Nototheniidae	*Trematomus bernacchii*	YPM ICH 22400
Nototheniidae	*Trematomus bernacchii*	YPM ICH 16652
Nototheniidae	*Trematomus borchgrevinki*	TA537PAB01
Nototheniidae	*Trematomus eulepidotus*	YPM ICH 16496
Nototheniidae	*Trematomus eulepidotus*	YPM ICH 16496
Nototheniidae	*Trematomus eulepidotus*	YPM ICH 16496
Nototheniidae	*Trematomus hansoni*	YPM ICH 20463
Nototheniidae	*Trematomus hansoni*	YPM ICH 22685
Nototheniidae	*Trematomus hansoni*	YPM ICH 22685
Nototheniidae	*Trematomus lepidorhinus*	NMNZ P057959
Nototheniidae	*Trematomus lepidorhinus*	NMNZ P043658
Nototheniidae	*Trematomus lepidorhinus*	NMNZ P043594
Nototheniidae	*Trematomus loennbergii*	YPM ICH 20018
Nototheniidae	*Trematomus loennbergii*	YPM ICH 20019
Nototheniidae	*Trematomus loennbergii*	YPM ICH 20020
Nototheniidae	*Trematomus newnesi*	YPM ICH 20492
Nototheniidae	*Trematomus newnesi*	YPM ICH 20492
Nototheniidae	*Trematomus newnesi*	YPM ICH 20492
Nototheniidae	*Trematomus nicolai*	YPM ICH 18359
Nototheniidae	*Trematomus nicolai*	YPM ICH 18359
Nototheniidae	*Trematomus nicolai*	YPM ICH 18359
Nototheniidae	*Trematomus pennellii*	YPM ICH 24242
Nototheniidae	*Trematomus pennellii*	YPM ICH 24242
Nototheniidae	*Trematomus pennellii*	YPM ICH 16507
Nototheniidae	*Trematomus scotti*	YPM ICH 24279
Nototheniidae	*Trematomus scotti*	YPM ICH 24279
Nototheniidae	*Trematomus scotti*	YPM ICH 24279
Nototheniidae	*Trematomus tokarevi*	YPM ICH 22481
Nototheniidae	*Trematomus tokarevi*	YPM ICH 20027
Nototheniidae	*Trematomus tokarevi*	YPM ICH 22408

Table S2. Genus, species, abbreviations, and common names in Figures 4 and S1-S5.

Genus species	Abbreviation	Group[2]	Common name[3]
Anarrhichthys ocellatus	*Anaoce*	P	wolf eel
Chaenocephalus aceratus	*Chaace*	C	blackfin icefish
Chaenodraco wilsoni	*Chawil*	C	spiny icefish
Champsocephalus esox	*Chaeso*	C	pike icefish
Champsocephalus gunnari	*Chagun*	C	mackerel icefish
Chionobathyscus dewitti	*Chidew*	C	NA
Chionodraco hamatus	*Chiham*	C	NA
Chionodraco myersi	*Chimye*	C	Myers' icefish
Chionodraco rastrospinosus	*Chiras*	C	ocellated icefish
Cottoperca gobio	*Cotgob*	N	channel bull blenny
Danio rerio	*Danrer*	T	zebrafish
Dissostichus mawsoni	*Dismaw*	C	Antarctic toothfish
Dolloidraco longedorsalis	*Dollon*	C	NA
Eleginops maclovinus	*Elemac*	N	Patagonian blennie
Epinephelus lanceolatus	*Epilan*	P	giant grouper
Gobionotothen gibberifrons	*Gobgib*	C	humped rockcod
Gymnodraco acuticeps	*Gymacu*	C	ploughfish
Harpagifer antarcticus	*Harant*	C	Antarctic spiny plunderfish
Notothenia angustata	*Notang*	C	Maori chief
Notothenia coriiceps	*Notcor*	C	black rockcod
Nototheniops nudifrons	*Notnud*	C	yellowfin notie
Pagetopsis macropterus	*Pagmac*	C	NA
Pseudochaenichthys georgianus	*Psegeo*	C	South Georgia icefish
Psilodraco breviceps	*Psibre*	C	NA
Trematomus bernacchii	*Treber*	C	emerald rockcod
Trematomus borchgrevinki	*Trebor*	C	bald notothen
Trematomus loennbergii	*Treloe*	C	Scaly rockcod
Trematomus newnesi	*Trenew*	C	Dusky rockcod

[2] Groups: C = cryonotothenioid ice fish; N = non-ice fish notothenioid; P = perciform outgroup; T = teleost outgroup
[3] Common names from FishBase (https://www.fishbase.se/home.php)

Table S3. Opsin sequence identifiers included in Figures 4 and S1-S5.

Species	Rh1[4]	Rh2	SWS1	SWS2	LWS	References
Anaoce	XP_031700525	XP_031708683<br>XP_031698504	XP_031696717	XP_031728565<br>XP_031728434	XP_033482814	
Chaace	HQ170035<br>HQ170036					
Chawil	HQ170037					
Chaeso	HQ170040					
Chagun	HQ170041		BAJ15894*		BAL15673**	*Miyazaki et al 2011;<br>**Miyazaki and Iwami, 2012
Chidew	HQ170045					
Chiham	HQ170047					
Chimye	HQ170048<br>(HQ170049)					
Chiras	HQ170050<br>(HQ170051)					
Cotgob	XP_029291931*	XP_029286851*<br>XP_029286846*	XP_029282297*	XP_029288360*<br>XP_029288359*	XP_029287453*	*Bista et al 2020
Danrer	BAC21668**<br>ADP06864***	BAC24129*<br>BAC24130*<br>BAC24131*<br>BAC24132*	BAC24134*	BAC24133*	BAC24127*<br>BAC24128*	*Chinen et al 2002;<br>**Hamaoka et al 2002;<br>***Morrow et al. 2017
Dismaw	DQ498794***	KAF3856251*<br>AAV53958**	AAX99140**			*Jae Lee et al 2021<br>**Pointer et al. 2005<br>***Sanchez et al. 2007
Dollon	HQ170034					
Elemac	AY141303***	evm.model.<br>scaffold423.2*	evm.model.<br>scaffold79.26*<br>(partial)	AJD38779**<br>AJD38778**	evm.model.<br>scaffold383.27*<br>(pseudogene?)	*Chen et al 2019<br>**Cortesi et al<br>***Sanchez et al. 2007
Epilan	XP_033484724*	XP_033483237*<br>XP_033483238*<br>XP_033483239*	XP_033470999*	XP_033482114*<br>XP_033482113*<br>XP_033483640*	XP_033482814*	*Wang et al 2019
Gobgib					BAL15677*	*Miyazaki and Iwami, 2012
Gymacu	XP_034066827*	XP_034075023*<br>XP_034075029*<br>AAV53961**	XP_034055527*<br>AAX99145**	XP_034068225*	XP_034068146*	*NCBI Genome Annotation Release 100<br>**Pointer et al. 2005
Harant	HQ170033					
Notang	DQ498787**	AAV53960*	AAX99143*			*Pointer et al. 2005<br>**Sanchez et al. 2007
Notcor	XP_010779059*	XP_010780753*		XP_010769741*<br>(pseudogene?)	XP_010769740*	*Lin et al, 2017 and Shin et al, 2014
Notnud	HM166262				BAL15676*<br>BAL15680*	*Miyazaki and Iwami, 2012
Pagmac	EU637990*<br>(HQ170061)		AAX99144**			*Li et al, 2009<br>**Pointer et al. 2005
Psegeo	XP_033942519*	XP_033938873*<br>XP_033938753*	XP_033935414*	XP_033939966*	XP_033938770*	*NCBI Genome Annotation Release 100
Psibre	AMW90782<br>(KU647484)					
Treber	XP_033982463*	XP_033994711*<br>XP_033994756*	XP_033973103*	XP_033977579*<br>XP_033977564*	XP_033977647*	*NCBI Genome Annotation Release 100
Trebor	HM166264	AAV53959*	AAX99142*			*Pointer et al. 2005
Tremloe	HM166275<br>DQ498802**		AAX99141*	AAV53962*		*Pointer et al. 2005<br>**Sanchez et al. 2007
Trenew	HM166276				BAL15675*	*Miyazaki and Iwami, 2012

[4] Sequences that encode identical proteins are listed in parentheses and excluded from analyses.

Table S4. Changes in major tuning sites and their predicted λmax[5].

	Rh1[6]			Rh2[7]		SWS2[8]	SWS1[9]		LWS[10]	
Species	D83N ↓3 nm	E122Q ↓13 nm	A292S ↓2 nm	E122Q ↓13-17 nm	M207L ↓6-10 nm	W265Y ↓29 nm	S114A	S118A ↓5 nm	S164A ↓7 nm	S164P
Chaace	2/2	0/2	0/2	0/0	0/0	0/0	0/0	0/0	0/0	0/0
Chawil	1/1	0/1	0/1	0/0	0/0	0/0	0/0	0/0	0/0	0/0
Chaeso	0/1	0/1	0/1	0/0	0/0	0/0	0/0	0/0	0/0	0/0
Chagun	1/1	0/1	0/1	0/0	0/0	0/0	1/1	1/1	1/1	0/1
Chidew	1/1	0/1	1/1	0/0	0/0	0/0	0/0	0/0	0/0	0/0
Chiham	1/1	0/1	0/1	0/0	0/0	0/0	0/0	0/0	0/0	0/0
Chimye	1/1	0/1	0/1	0/0	0/0	0/0	0/0	0/0	0/0	0/0
Chiras	1/1	0/1	0/1	0/0	0/0	0/0	0/0	0/0	0/0	0/0
Dismaw	0/1	0/1	0/1	2/2	1/2	0/0	0/1	0/1	0/0	0/0
Dollon	0/1	0/1	1/1	0/0	0/0	0/0	0/0	0/0	0/0	0/0
Gobgib	0/0	0/0	0/0	0/0	0/0	0/0	0/0	0/0	1/1	0/1
Gymacu	1/1	0/1	1/1	3/3	3/3	0/1	2/2	0/2	1/1	0/1
Harant	0/1	0/1	0/1	0/0	0/0	0/0	0/0	0/0	0/0	0/0
Notang	0/1	0/1	0/1	0/1	0/1	0/0	1/1	1/1	0/0	0/0
Notcor	0/1	1/1	0/1	1/1	0/1	0/1	0/0	0/0	0/1	0/1
Notnud	0/1	1/1	0/1	0/0	0/0	0/0	0/0	0/0	2/2	0/2
Pagmac	1/1	0/1	0/1	0/0	0/0	0/0	0/1	0/1	0/0	0/0
Psegeo	1/1	0/1	0/1	2/2	2/2	1/1	0/1	0/1	1/1	0/1
Psibre	1/1	0/1	0/1	0/0	0/0	0/0	0/0	0/0	0/0	0/0
Treber	0/1	0/1	0/1	2/2	2/2	0/0	0/1	0/1	0/1	1/1
Trebor	0/1	0/1	0/1	1/1	1/1	1/2	0/1	0/1	0/0	0/0
Treloe	0/2	0/2	021	0/0	0/0	0/1	1/1	0/1	0/0	0/0
Trenew	0/1	0/1	0/1	0/0	0/0	0/0	0/0	0/0	1/1	0/1

[5] Numbers indicate the number of sequences with indicated tuning site change / total number of sequences identified. Blue shading indicates predicted decreases in λ_{max} and orange shading indicates an unknown impact on λ_{max}. Gray shading indicates that no sequences were identified.
[6] Predicted shifts in λ_{max} of Rh1 from Yokoyama et al (2008a). Note that the combination of D83N and A292S can decrease λ_{max} by 14-17 nm. Changes in other major tuning site F261 (Lin et al 2017) were not observed.
[7] Predicted shifts in λ_{max} of Rh2 from Yokoyama et al (1999), Takenaka and Yokoyama (2007) and Yokoyama and Jia (2020). Changes in other major tuning sites S97 and A292 (Lin et al 2017) were not observed.
[8] Predicted shifts in λ_{max} of SWS2 from Yokoyama et al (2007). Changes in other major tuning sites A94, S97, and T118, (Lin et al 2017) were not observed.
[9] Shi and Yokoyama (2003) demonstrated the reverse mutation A118S increases λ_{max} of SWS1 by 5 nm, thus we predict a comparable decrease for S118A. Changes in other major tuning sites T52, F86, and Q93 (Lin et al 2017) were not observed.
[10] Predicted shift for LWS S164A from Yokoyama et al (2008b). Changes in other major tuning sites H181, Y261, T269, and A292 (Lin et al 2017) were not observed.

Table S5. Results from PGLS regressions of residual eye size (based on HL) on depth and buoyancy for all notothenioid species.

Predictor Variable	Parameter estimate (β) ± SE	t-value	*p*-value[11]
Mean depth of occurrence	0.00016 ± 0.0012	0.13	0.90
Minimum reported depth	-0.00080 ± 0.00088	-0.91	0.90
Maximum reported depth	-0.000078 ± 0.00035	-0.22	0.90
Mean % Buoyancy	-0.097 ± 0.18	-0.53	0.90

11. P-values have been adjusted to correct for multiple comparisons using the false discovery rate correction method of Benjamini and Hochberg (1995).

Table S6. Results of tests of phylogenetic signal in residual eye size relative to head size.

	Pagel's lambda		Blomberg et al.'s K	
Trait	Estimate	*p*-value	Estimate	*p*-value
Residual Eye Size	0.36	**0.001**	0.35	0.27

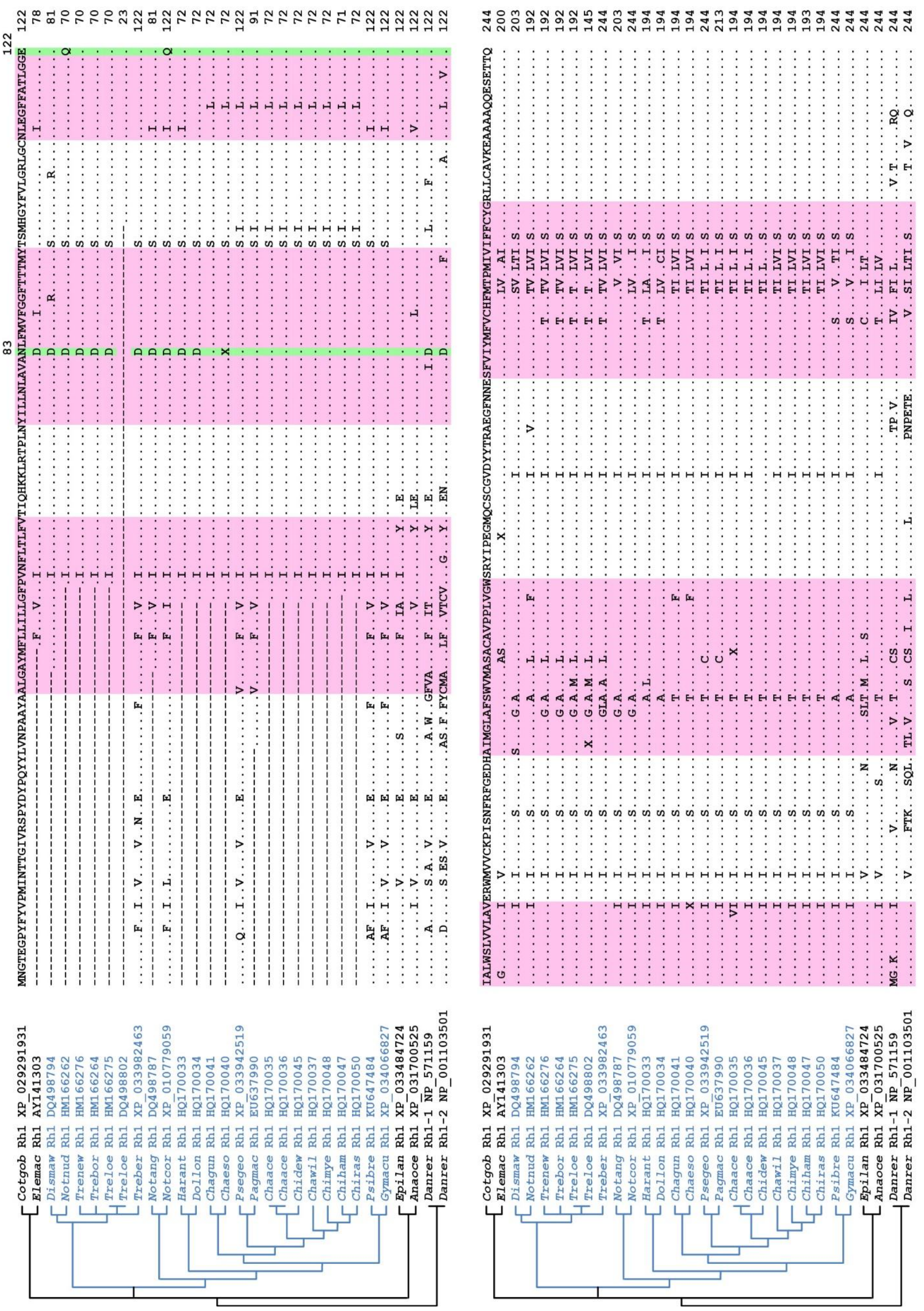
Cotgob Rh1 XP_029291931
Elemac Rh1 AY141303
Dismaw Rh1 DQ498794
Notnud Rh1 HM166262
Trenew Rh1 HM166276
Trebor Rh1 HM166264
Treloe Rh1 HM166275
Treloe Rh1 DQ498802
Treber Rh1 XP_033982463
Notang Rh1 DQ498787
Notcor Rh1 XP_010779059
Harant Rh1 HQ170033
Dollon Rh1 HQ170034
Chagun Rh1 HQ170041
Chaeso Rh1 HQ170040
Psegeo Rh1 XP_033942519
Pagmac Rh1 EU637990
Chaace Rh1 HQ170035
Chaace Rh1 HQ170036
Chidew Rh1 HQ170045
Chawil Rh1 HQ170037
Chimye Rh1 HQ170048
Chiham Rh1 HQ170047
Chiras Rh1 HQ170050
Psibre Rh1 KU647484
Gymacu Rh1 XP_034066827
Epilan Rh1 XP_033484724
Anaoce Rh1 XP_031700525
Danrer Rh1-1 NP_571159
Danrer Rh1-2 NP_001103501
83
122

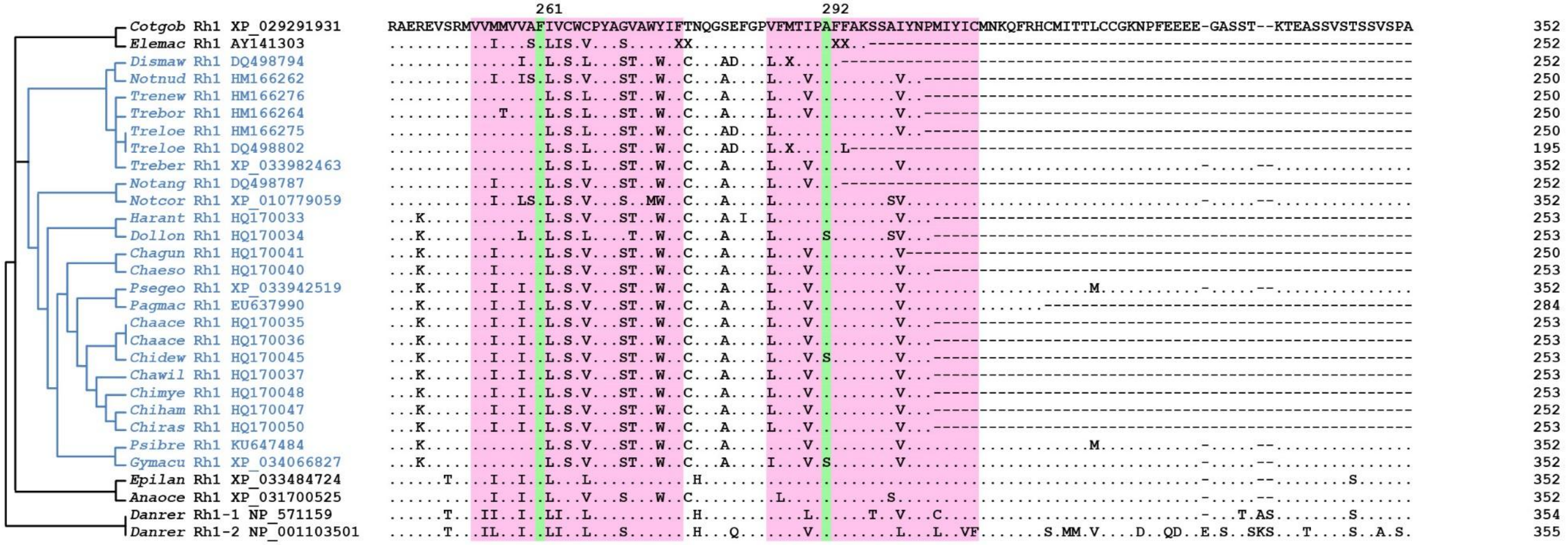

Figure S1. Notothenioid Rh1 sequences and tuning sites.

Alignment of representative cryonotothenioid Rh1 protein sequences compared with representative non-icefish notothenioid (*Cottoperca gobio, Cotgob and Eleginops maclovinus, Elemac*), perciforme (*Anarrhichthys ocellatus*, *Anaoce* and *Epinephelus lanceolatus, Epilan*) and zebrafish (*Danio rerio, Danrer*) Rh1 sequences. The phylogenetic trees (left) show the relationship between icefish (blue text), other perciformes, and zebrafish. The *Cotgob* sequence was used as a reference (top row) and dots in the alignment indicate identity. Differences from *Cotgob* are indicated by the appropriate amino acid symbol. Amino acids in transmembrane domains are highlighted pink. Major tuning site amino acids (Lin et al., 2017) are highlighted green with the position number of the amino acid at the top of each column. Position numbers are based on the bovine rhodopsin (Palczewski et al, 2000). Sequence identifiers are included in the alignment and listed in Supplemental Table S2. Genus and species are included in Supplemental Table S1.

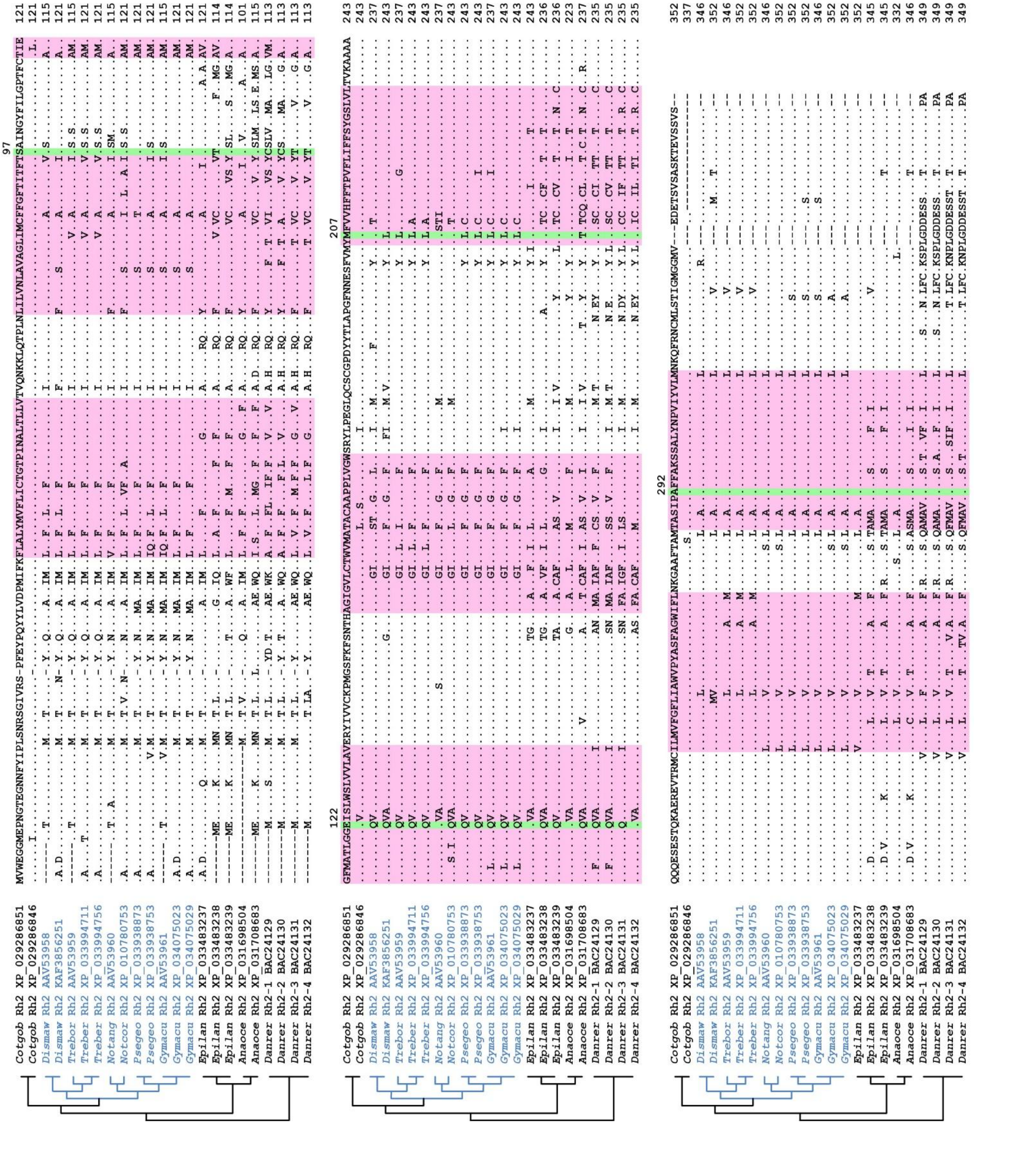

Figure S2. Notothenioid Rh2 sequences and tuning sites.

Alignment of representative cryonotothenioid Rh2 protein sequences compared with representative non-icefish notothenioid (*Cottoperca gobio, Cotgob*), perciforme (*Anarrhichthys ocellatus, Anaoce* and *Epinephelus lanceolatus, Epilan*) and zebrafish (*Danio rerio, Danrer*) Rh2 sequences. The phylogenetic trees (left) show the relationship between icefish (blue text), other perciformes, and zebrafish. A *Cotgob* sequence was used as a reference (top row) and dots in the alignment indicate identity. Differences from *Cotgob* are indicated by the appropriate amino acid symbol. Amino acids in transmembrane domains are highlighted pink. Major tuning site amino acids (Lin et al., 2017) are highlighted green with the position number of the amino acid at the top of each column. Position numbers are based on bovine rhodopsin (Palczewski et al, 2000). Sequence identifiers are included in the alignment and listed in Supplemental Table S2. Genus and species are included in Supplemental Table S1.

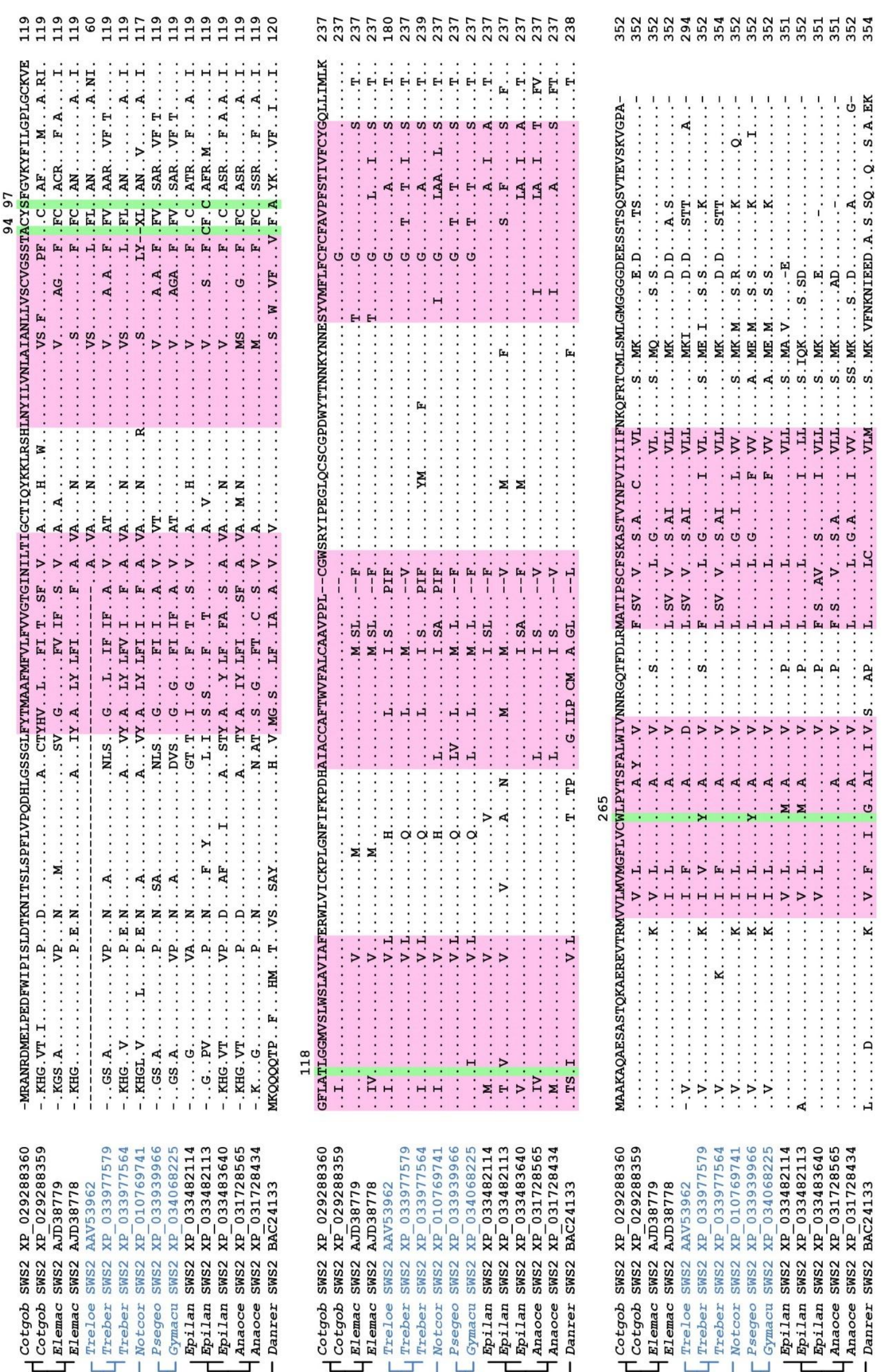
Cotgob SWS2 XP_029288360
Cotgob SWS2 XP_029288359
Elemac SWS2 AJD38779
Elemac SWS2 AJD38778
Treloe SWS2 AAV53962
Treber SWS2 XP_033977579
Treber SWS2 XP_033977564
Notcor SWS2 XP_010769741
Psegeo SWS2 XP_033939966
Gymacu SWS2 XP_034068225
Epilan SWS2 XP_033482114
Epilan SWS2 XP_033482113
Epilan SWS2 XP_033483640
Anaoce SWS2 XP_031728565
Anaoce SWS2 XP_031728434
Danrer SWS2 BAC24133
94
97
118
265

Figure S3. Notothenioid SWS2 sequences and tuning sites.

Alignment of representative cryonotothenioid SWS2 protein sequences compared with representative non-icefish notothenioid (*Cottoperca gobio, Cotgob* and *Eleginops maclovinus, Elemac*), perciforme (*Anarrhichthys ocellatus, Anaoce* and *Epinephelus lanceolatus, Epilan*) and zebrafish (*Danio rerio, Danrer*) SWS2 sequences. The phylogenetic trees (left) show the relationship between icefish (blue text), other perciformes, and zebrafish. A *Cotgob* sequence was used as a reference (top row) and dots in the alignment indicate identity. Differences from *Cotgob* are indicated by the appropriate amino acid symbol. Amino acids in transmembrane domains are highlighted pink. Major tuning site amino acids (Lin et al., 2017) are highlighted green with the position number of the amino acid at the top of each column. Position numbers are based on bovine rhodopsin (Palczewski et al, 2000). Sequence identifiers are included in the alignment and listed in Supplemental Table S2. Genus and species are included in Supplemental Table S1.

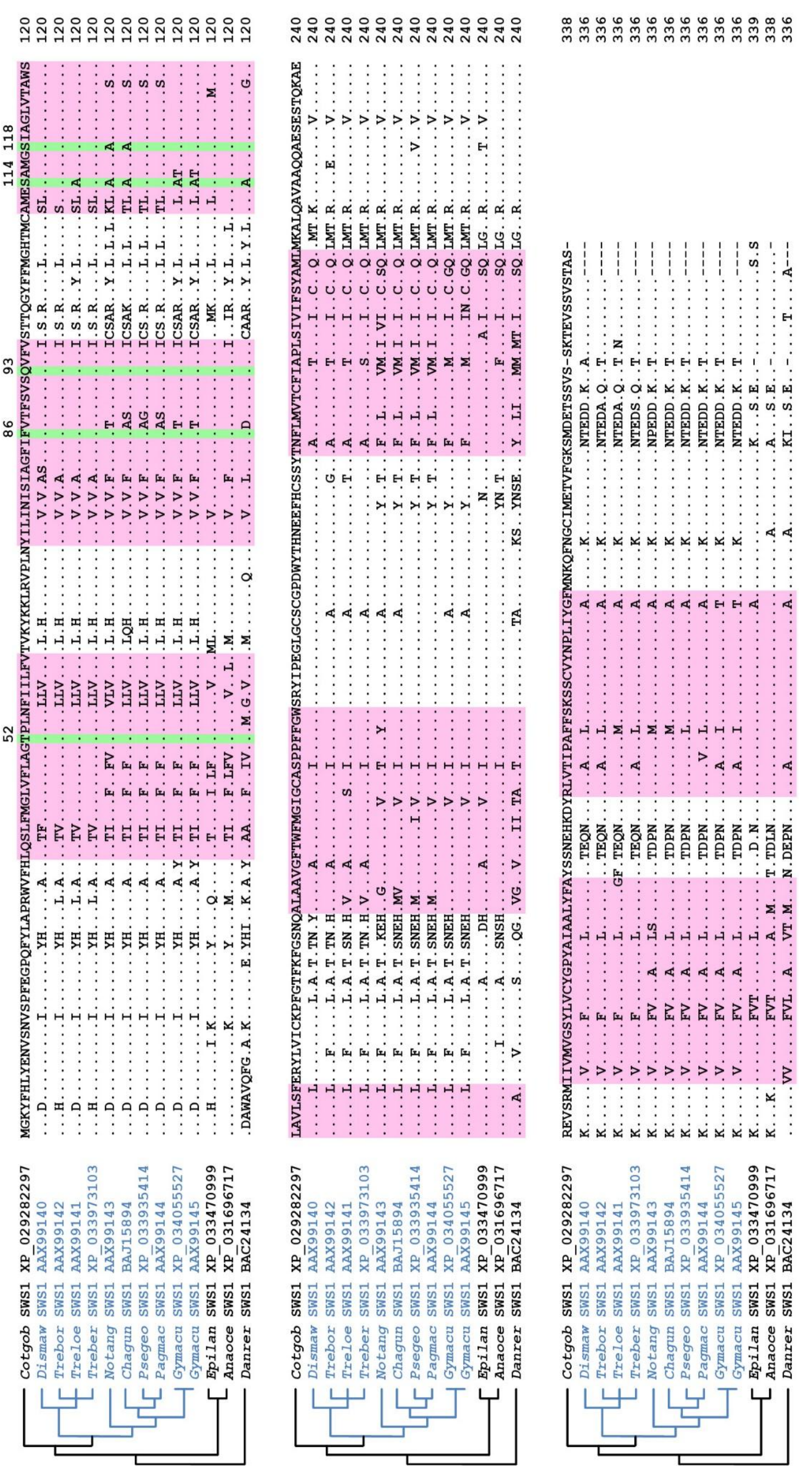
Cotgob SWS1 XP_029282297
Dismaw SWS1 AAX99140
Trebor SWS1 AAX99142
Treloe SWS1 AAX99141
Treber SWS1 XP_033973103
Notang SWS1 AAX99143
Chagun SWS1 BAJ15894
Psegeo SWS1 XP_033935414
Pagmac SWS1 AAX99144
Gymacu SWS1 XP_034055527
Gymacu SWS1 AAX99145
Epilan SWS1 XP_033470999
Anaoce SWS1 XP_031696717
Danrer SWS1 BAC24134
52
86
93
114 118

Figure S4. Notothenioid SWS1 sequences and tuning sites.

Alignment of representative cryonotothenioid SWS1 protein sequences compared with representative non-icefish notothenioid (*Cottoperca gobio, Cotgob*), perciforme (*Anarrhichthys ocellatus*, *Anaoce* and *Epinephelus lanceolatus, Epilan*) and zebrafish (*Danio rerio, Danrer*) SWS1 sequences. The phylogenetic trees (left) show the relationship between icefish (blue text), other perciformes, and zebrafish. The *Cotgob* sequence was used as a reference (top row) and dots in the alignment indicate identity. Differences from *Cotgob* are indicated by the appropriate amino acid symbol. Amino acids in transmembrane domains are highlighted pink. Major tuning site amino acids (Lin et al., 2017) are highlighted green with the position number of the amino acid at the top of each column. Position numbers are based on bovine rhodopsin (Palczewski et al, 2000). Sequence identifiers are included in the alignment and listed in Supplemental Table S2. Genus and species are included in Supplemental Table S1.

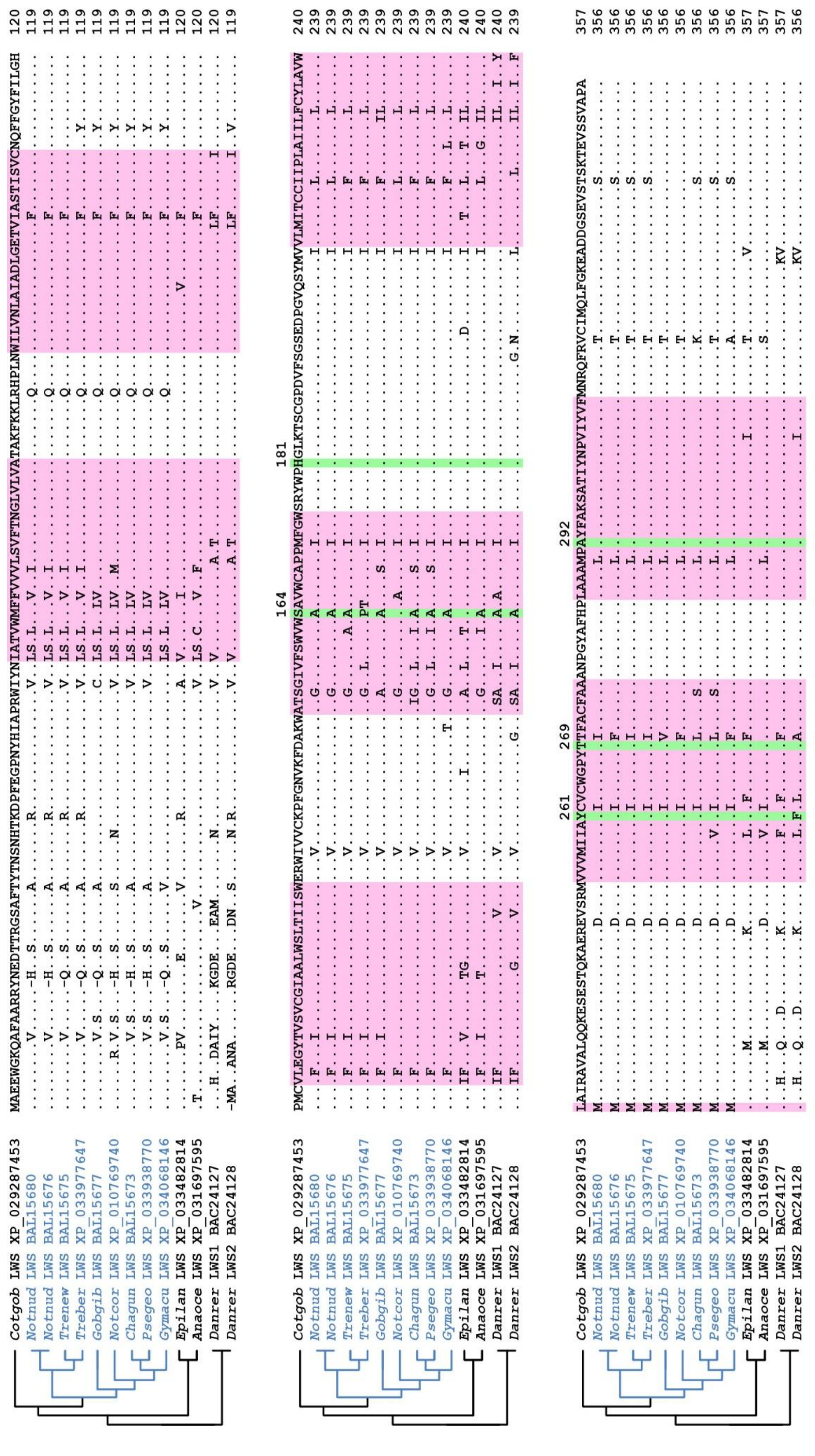
Cotgob LWS XP_029287453
Notnud LWS BAL15680
Notnud LWS BAL15676
Trenew LWS BAL15675
Treber LWS XP_033977647
Gobgib LWS BAL15677
Notcor LWS XP_010769740
Chagun LWS BAL15673
Psegeo LWS XP_033938770
Gymacu LWS XP_034068146
Epilan LWS XP_033482814
Anaoce LWS XP_031697595
Danrer LWS1 BAC24127
Danrer LWS2 BAC24128
164
181
261
269
292

Figure S5. Notothenioid LWS sequences and tuning sites.

Alignment of representative cryonotothenioid LWS protein sequences compared with representative non-icefish notothenioid (*Cottoperca gobio, Cotgob*), perciforme (*Anarrhichthys ocellatus*, *Anaoce* and *Epinephelus lanceolatus, Epilan*) and zebrafish (*Danio rerio, Danrer*) LWS sequences. The phylogenetic trees (left) show the relationship between icefish (blue text), other perciformes, and zebrafish. The *Cotgob* sequence was used as a reference (top row) and dots in the alignment indicate identity. Differences from *Cotgob* are indicated by the appropriate amino acid symbol. Amino acids in transmembrane domains are highlighted pink. Major tuning site amino acids (Lin et al., 2017) are highlighted green with the position number of the amino acid at the top of each column. Position numbers are based on bovine rhodopsin (Palczewski et al, 2000). Sequence identifiers are included in the alignment and listed in Supplemental Table S2. Genus and species are included in Supplemental Table S1.

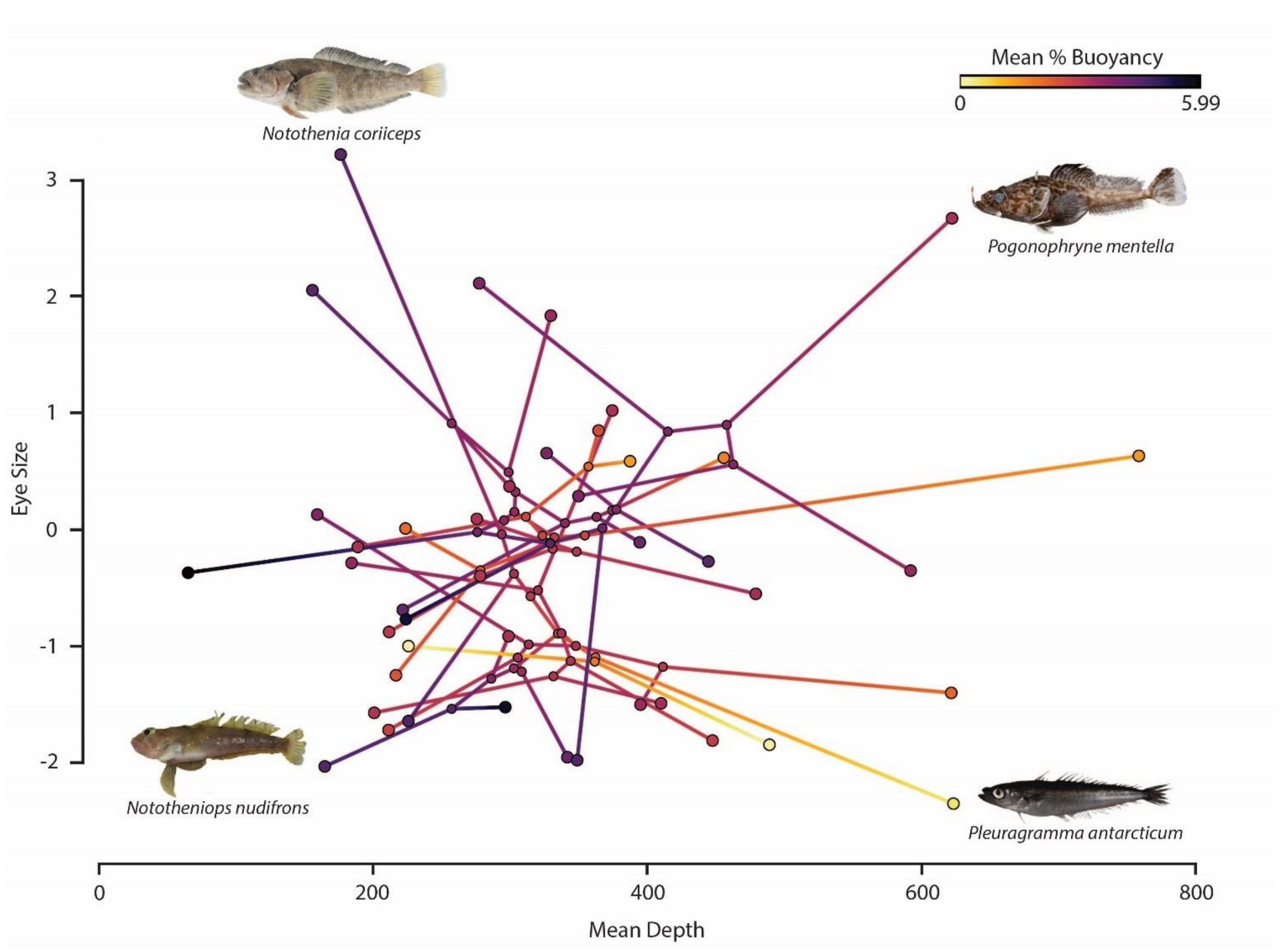

Figure S6. Phylomorphospace plot of depth versus residual eye size.

Phylomorphospace depicting projection of notothenioid phylogeny into trait space defined by mean depth of occurrence on the X axis and relative eye size on the Y axis. Larger shaded circles correspond to each of the notothenioid species sampled in our phylogeny, while smaller shaded circles correspond to nodes in the phylogeny. The ancestral state reconstruction of mean percentage buoyancy (% B) has been mapped onto the projected phylogeny to facilitate simultaneous visualization of evolutionary changes in mean depth of occurrence, eye size, and buoyancy.

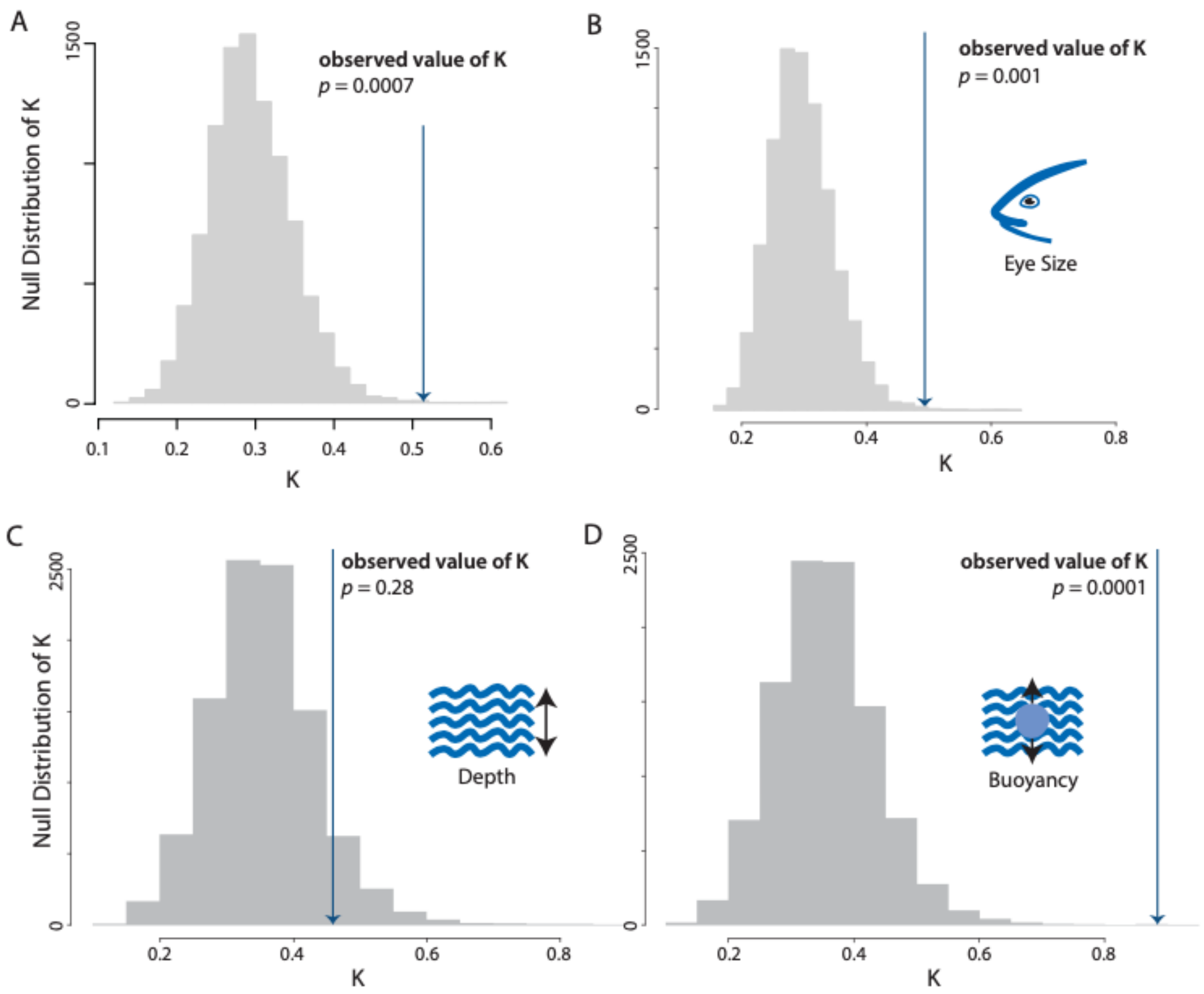

Figure S7. Phylogenetic signal in ecomorphological traits.

Comparisons of empirical values of Blomberg et al.'s K (2003) calculated for residual eye size relative to SL (A), residual eye size relative to HL (B), mean depth of occurrence (C), and mean percentage buoyancy (D) to distribution of K values calculated from 10,000 permutations of trait evolution on the notothenioid phylogeny.

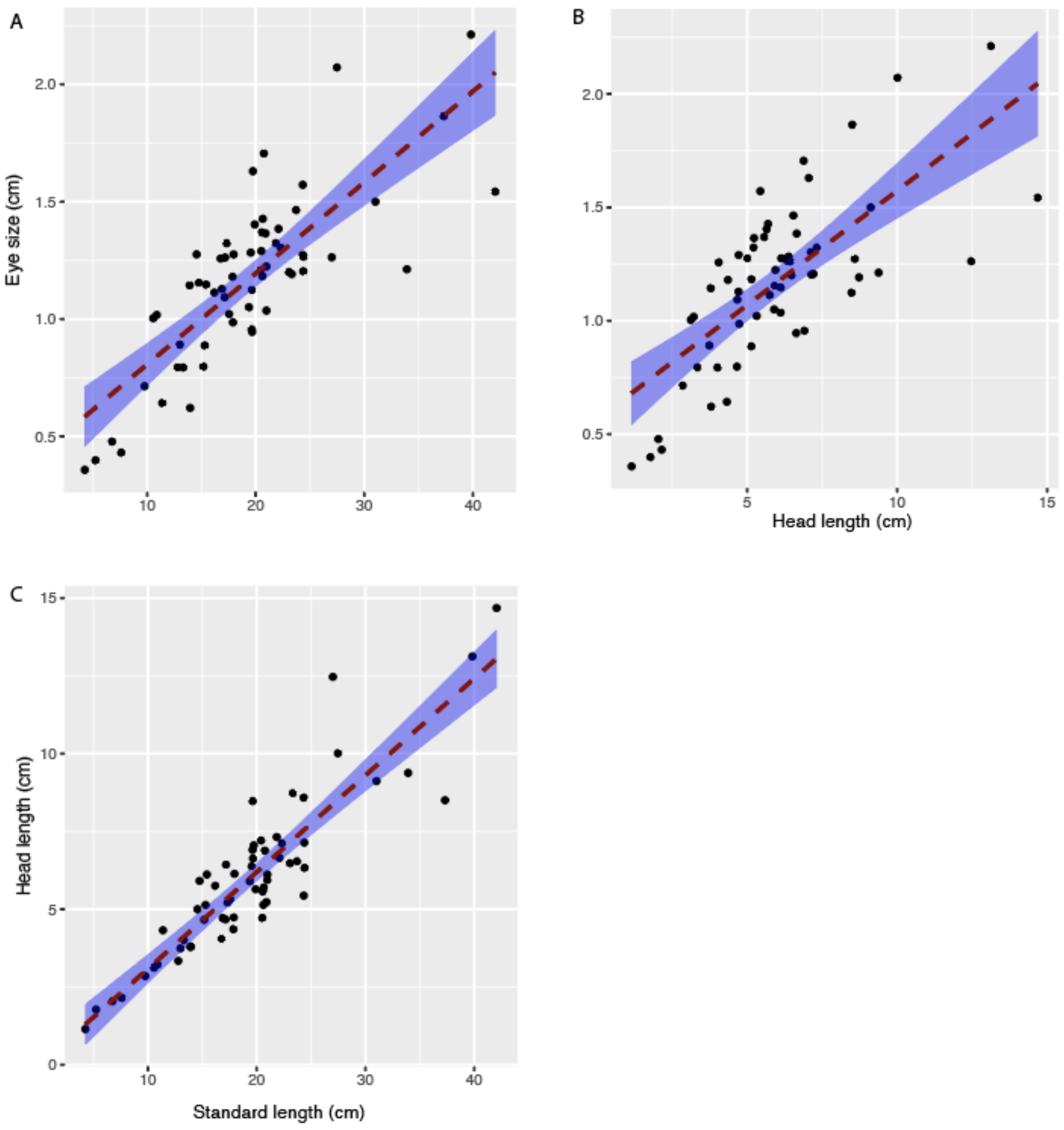

Figure S8. Correlation between morphological traits.

Visualizations of the phylogenetic regressions for eye size~SL (A), eye size~HL (B), and HL~SL (C). In all cases $p < 0.0001$.

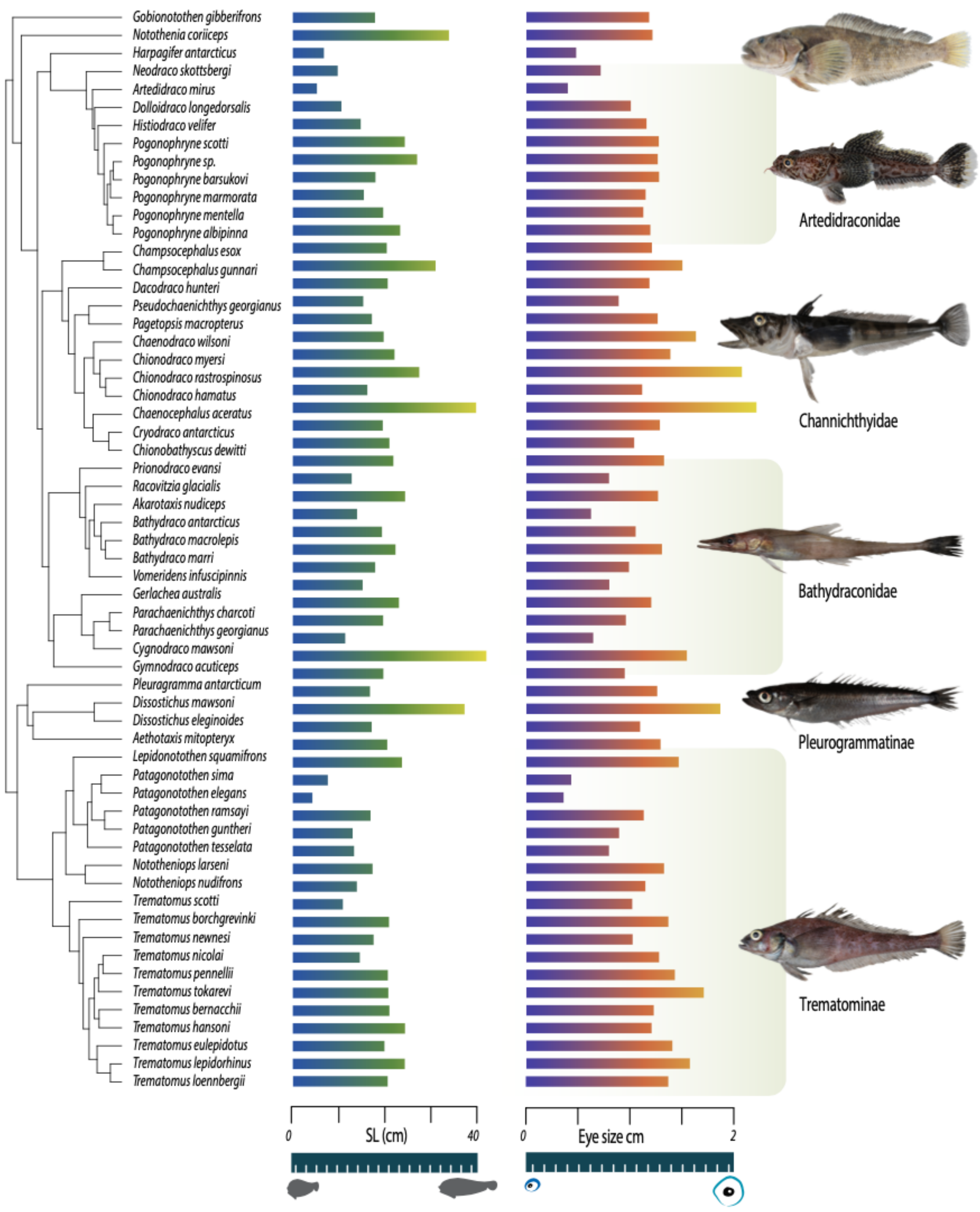

Figure S9. Visualization of morphological measurements.

Shown on the left panel is a time-calibrated tree depicting phylogenetic relationships among notothenioid species sampled in our morphological dataset. The middle panel depicts average standard length measured per species, with darker shadings corresponding to smaller standard lengths. The right panel depicts a barplot of average eye sizes measured for each species with warm colors representing larger eyes. Fish images: EP.

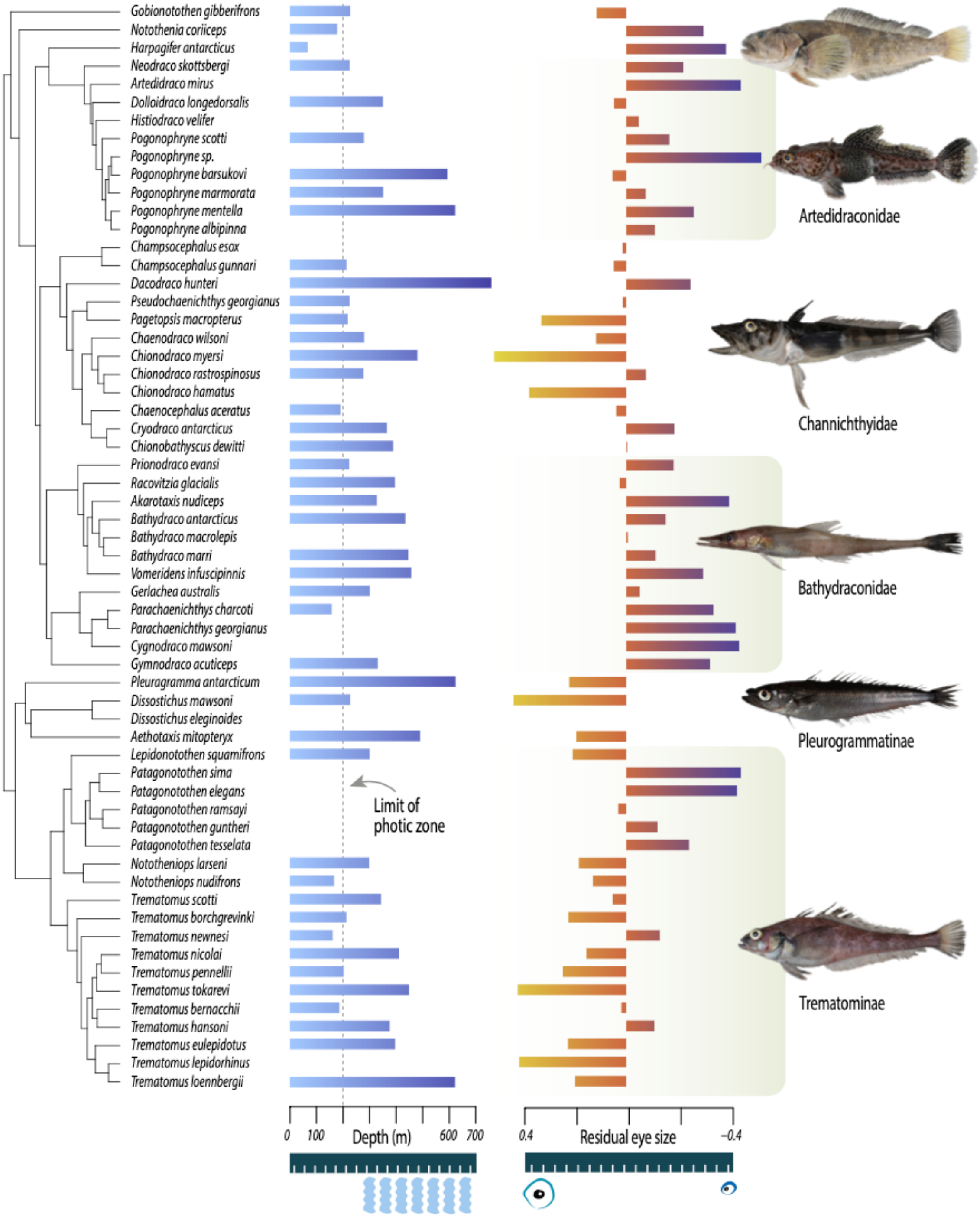

Figure S10. Visualization of variation in mean depth and eye size across the notothenioid phylogeny.
Shown on the left panel is a time-calibrated tree depicting phylogenetic relationships among notothenioid species sampled in our morphological dataset. The middle panel depicts average depth per species, with darker shadings corresponding to deeper depths. The right panel depicts a barplot of eye size (represented as residuals from the regression of eye diameter on head length) measured for our focal notothenioid species with warm colors representing larger eyes relative to head size. Fish images: EP.

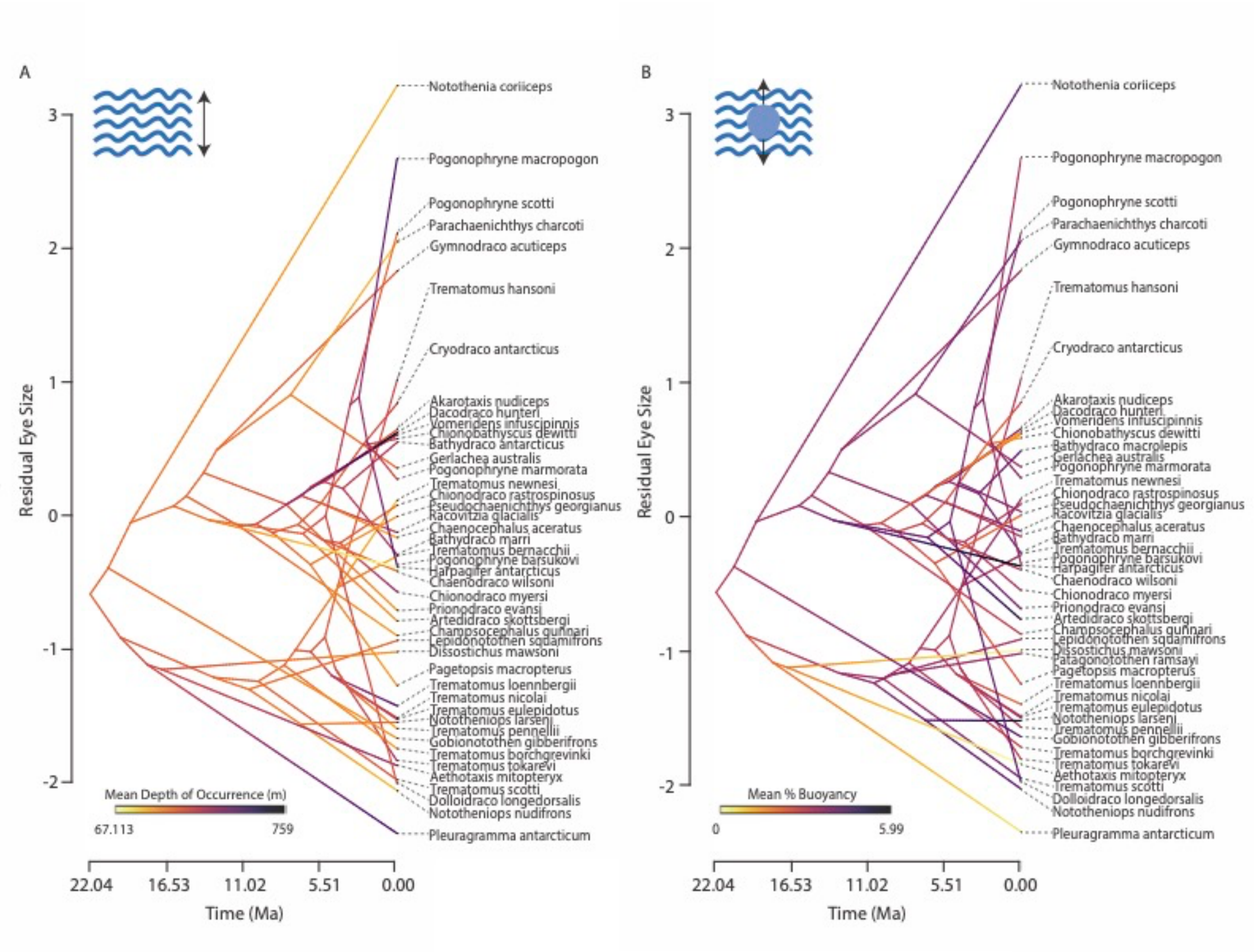

Figure S11. Projection of notothenioid phylogeny in space defined by time and residual variation in eye size.

Time (in millions of years) is on the X axis and eye size variability is on the Y axis. Placement of tree tips along the Y axis corresponds to eye size (represented using the residuals from regression of eye diameter on head length) for each notothenioid species. Ancestral state reconstructions of mean depth of occurrence (panel A) and of mean %B (panel B) have been mapped onto the notothenioid phylogeny to facilitate simultaneous visualization of variation in eye size and variation in water column usage.

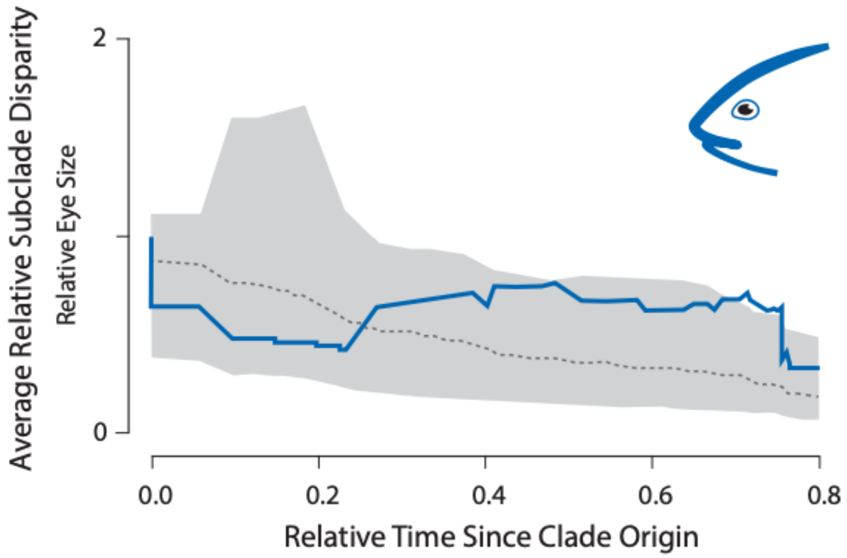

Figure S12. Disparity through time (DTT; Harmon et al. 2003) over the course of the notothenioid radiation.

Graph depicts patterns of disparity in residual eye size corrected for head length. The X axis reflects relative time since clade origin (0.0). The Y axis corresponds to average relative subclade disparity in eye size. The solid blue line depicts the empirical estimation of eye size disparity, while the dotted gray line depicts the median trait disparity calculated from 10,000 Brownian motion simulations of trait evolution on the notothenioid phylogeny. The shaded gray region represents the 95% confidence interval (CI) of the Brownian motion simulations.

References.

Bista I, McCarthy SA, Wood J, Ning Z, Detrich Iii HW, Desvignes T, Postlethwait J, Chow W, Howe K, Torrance J, Smith M, Oliver K; Vertebrate Genomes Project Consortium, Miska EA, Durbin R. 2020. The genome sequence of the channel bull blenny, Cottoperca gobio (Günther, 1861). *Wellcome Open Res*. 24; 5:148.

Blomberg SP, Garland T, Ives AR. 2003. Testing for phylogenetic signal in comparative data: behavioral traits are more labile. Evolution. 57(4): 717-45.

Chen L, Lu Y, Li W, Ren Y, Yu M, Jiang S, Fu Y, Wang J, Peng S, Bilyk KT, Murphy KR, Zhuang X, Hune M, Zhai W, Wang W, Xu Q, Cheng CC. 2019. The genomic basis for colonizing the freezing Southern Ocean revealed by Antarctic toothfish and Patagonian robalo genomes. *Gigascience*. 8(4): giz016.

Chinen A, Hamaoka T, Yamada Y, Kawamura S. 2003. Gene duplication and spectral diversification of cone visual pigments of zebrafish. *Genetics*. 163(2): 663-75.

Cortesi F, Musilová Z, Stieb SM, Hart NS, Siebeck UE, Malmstrøm M, Tørresen OK, Jentoft S, Cheney KL, Marshall NJ, Carleton KL, Salzburger W. 2015. Ancestral duplications and highly dynamic opsin gene evolution in percomorph fishes. *Proc Natl Acad Sci U S A*.112(5):1493-8.

Fujiyabu C, Sato K, Utari NML, Ohuchi H, Shichida Y, Yamashita T. 2019. Evolutionary history of teleost intron-containing and intron-less rhodopsin genes. *Sci Rep*. 9(1): 10653.

Hamaoka T, Takechi M, Chinen A, Nishiwaki Y, Kawamura S. 2002. Visualization of rod photoreceptor development using GFP-transgenic zebrafish. *Genesis*. 34(3): 215-20.

Jae Lee S, Kim JH, Jo E, Choi E, Kim J, Choi SG, Chung S, Kim HW, Park H. 2021. Chromosomal assembly of the Antarctic toothfish (Dissostichus mawsoni) genome using third-generation DNA sequencing and Hi-C technology. *Zool Res*. 42(1): 124-129.

Li B, Dettaï A, Cruaud C, Couloux A, Desoutter-Meniger M, Lecointre G. 2009. RNF213, a new nuclear marker for acanthomorph phylogeny. *Mol Phylogenet Evol*. 50(2): 345-63.

Lin JJ, Wang FY, Li WH, Wang TY. 2017. The rises and falls of opsin genes in 59 ray-finned fish genomes and their implications for environmental adaptation. *Sci Rep*. 7(1): 15568.

Miyazaki T, Iwami T. 2012. Molecular cloning of cDNA encoding red opsin gene in the retinas of five Antarctic notothenioid fishes. *Polar Biol*. 35: 775-783.

Miyazaki T, Nakata M, Kasagi S, Iwami T, Yamauchi M, Kawamura S. 2011. Molecular cloning of ultraviolet-sensitive visual pigment in juvenile Champsocephalus gunnari (Channichthyidae). *Polar Biol*. 34: 235-242.

Morrow JM, Lazic S, Dixon Fox M, Kuo C, Schott RK, de A Gutierrez E, Santini F, Tropepe V, Chang BS. 2017. A second visual rhodopsin gene, rh1-2, is expressed in zebrafish photoreceptors and found in other ray-finned fishes. *J Exp Biol*. 220(Pt 2): 294-303.

Palczewski K, Kumasaka T, Hori T, Behnke CA, Motoshima H, Fox BA, Le Trong I, Teller DC, Okada T, Stenkamp RE, Yamamoto M, Miyano M. 2000. Crystal structure of rhodopsin: A G protein-coupled receptor. *Science*. 289(5480): 739-45.

Pointer MA, Cheng CH, Bowmaker JK, Parry JW, Soto N, Jeffery G, Cowing JA, Hunt DM. 2005. Adaptations to an extreme environment: retinal organisation and spectral properties of photoreceptors in Antarctic notothenioid fish. *J Exp Biol*. 208(Pt 12): 2363-76.

Sabaj MH. 2016. Standard symbolic codes for institutional resource collections in herpetology and ichthyology: an Online Reference. Version 6.5 (16 August 2016). Electronically accessible at http://www.asih.org/, American Society of Ichthyologists and Herpetologists, Washington, DC.

Sanchez S, Dettai A, Bonillo C, Ozouf-Costaz C, Detrich III W, Lecointre G. 2007. Molecular and morphological phylogenies of the Antarctic teleostean family Nototheniidae, with emphasis on the Trematominae. *Polar Biol*. 30: 155-166.

Shin SC, Ahn DH, Kim SJ, Pyo CW, Lee H, Kim MK, Lee J, Lee JE, Detrich HW, Postlethwait JH, Edwards D, Lee SG, Lee JH, Park H. 2014. The genome sequence of the Antarctic bullhead notothen reveals evolutionary adaptations to a cold environment. *Genome Biol*. 15(9): 468.

Takenaka N, Yokoyama S. 2007. Mechanisms of spectral tuning in the RH2 pigments of Tokay gecko and American chameleon. *Gene*. 399(1): 26-32.

Wang D, Chen X, Zhang X, Li J, Yi Y, Bian C, Shi Q, Lin H, Li S, Zhang Y, You X. 2019. Whole Genome Sequencing of the Giant Grouper (Epinephelus lanceolatus) and High-Throughput Screening of Putative Antimicrobial Peptide Genes. *Mar Drugs*. 17(9): 503.

Yokoyama S, Jia H. 2020. Origin and adaptation of green-sensitive (RH2) pigments in vertebrates.

FEBS Open Bio. 10(5): 873-882.

Yokoyama S, Tada T, Zhang H, Britt L. 2008a. Elucidation of phenotypic adaptations: Molecular analyses of dim-light vision proteins in vertebrates. *Proc Natl Acad Sci U S A*. 105(36): 13480-5.

Yokoyama S, Takenaka N, Blow N. 2007. A novel spectral tuning in the short wavelength-sensitive (SWS1 and SWS2) pigments of bluefin killifish (*Lucania goodei*). *Gene*. 396(1): 196-202.

Yokoyama S, Yang H, Starmer WT. 2008b. Molecular basis of spectral tuning in the red- and green-sensitive (M/LWS) pigments in vertebrates. *Genetics*. 179(4): 2037-43.

Yokoyama S, Zhang H, Radlwimmer FB, Blow NS. 1999. Adaptive evolution of color vision of the Comoran coelacanth (*Latimeria chalumnae*). *Proc Natl Acad Sci U S A*. 96(11): 6279-84.
